# Supplementary material for: Development of New Antimicrobial Peptides by Directional Selection
Source: Antibiotics (Basel). 2025 Nov 6;14(11):1120. doi: 10.3390/antibiotics14111120 (PMC12649535; doi:10.3390/antibiotics14111120)
Supplement: Supplementary file 1 [file antibiotics-14-01120-s001.zip › antibiotics-3930108-supplementary.pdf]

Tables

Table S1. Antimicrobial activity of synthetic peptides against bacteria.

Peptide	Sequence, a.a.	MIC, mkM	
		E. coli	*B. subtilis*
cecropin	SWLSKTAKKLENSAKKRISEGIAIAIQGGPR	0.39	>50.00
CR1P5	SLMFKTVKKMDNSAKKRIREGIAIAIQGGPR	6.25	>50.00
CR2P2	SLMFITVKKLDNSSRIRIRVGIAIAIQGGPR	25.00	25.00
CR2P6	SLMFKTVKIPDNSPKKPIREVIAIAIQSGPR	>50.00	>50.00
CR2P7	SLIFKIVKKMDDIGKKRIREGIAIPLQGGPR	50.00	>50.00
CR2P8	SFVCKTVKKMNNSAKKGFREGIALAIQGGPR	6.25	25.00
CR2P9	SLMFKTVKKMDNSVKKRFREGMAIAMQCGPR	3.13	25.00
melittin	GIGAVLKVLTTGLPALISWIKRKRQQ	3.13	1.56
MR1P4	GIGAVLKVLTTCLTALISWIKRRRQQ	12.50	3.13
MR1P6	GIGAEFKVLSTCLSALISWLKRKRQQ	12.50	6.25
MR1P7	GIGALLKRLTPGLPTLISWIKRKCQL	3.13	1.56
MR1P8	GIGPLLKVLTTGHPALVSWIKCKRQQ	6.25	3.13
MR1P9	GIGAILKVQTTGLPALITWMKRIRQQ	12.50	3.13
Hm-AMP2	EKRWRRLIFNYF	3.13	12.50
A2R1P5	EKGWRRWIFNYF	6.25	6.25
A2R2P3	EKGWRRWIFYCF	50.00	25.00
A2R2P6	EIVWRRSIFNYF	>50.00	>50.00
A2R2P7	EIGWRRRICNYL	25.00	6.25
A2R3P6	EKGWRRWIFFSF	12.50	12.50
A2R3P7	EKGRRRWIFYFF	12.50	25.00
A2R3P8	EKGWLRRIFYCF	25.00	6.25
A2R3P9	ERGWFWRFFYYF	>50.00	>50.00
A2R3P10	EKDWPRRILYCF	>50.00	50.00
A2R3P11	EKGWSHWICQCL	>50.00	>50.00

Bacteria were treated for 24 h with peptides at a range of concentrations. The minimal inhibitory concentration (MIC) values were defined as the highest concentration that inhibited bacterial growth, as determined from three independent repetitions.

Table S2. Cytotoxic activity of cecropin mutants against Expi293F cells.

Concentration (mkM)/ peptide	cecropin	CR1P5	CR2P2	CR2P6	CR2P7	CR2P8	CR2P9	Control
200	79.3±10.8	79.4±5.9	36.3±12.9	74.8±11.6	52.2±6.8	72.8±7.6	56.1±14.6	-
100	96.1±4	93.3±3.9	88.4±7.3	88.4±10.1	81.7±11.4	86.2±11.0	94.8±3.4	-
50	94.7±6.2	93.8±2.5	94.9±5.5	95.1±5.2	91.7±3.9	95.9±3.3	93.4±2.4	-
25	89.3±10.2	94.3±3.3	95.7±6.2	94.3±2.3	95.2±1.7	95.1±3	94.4±2.0	-
12.5	89.9±5.6	97.0±1.5	95.6±3.2	93.2±2.3	94.7±2.2	94.7±2.7	94.9±2.6	-
6.25	90.2±7.4	94.2±3.7	93.5±2.1	95.8±2.4	94.3±2.6	9.06±2.6	95.9±3.1	-
3.13	92.0±3.2	95.0±2.8	91.8±3.9	94.4±2.4	95.6±2.1	95.2±3.7	95.0±2.5	-
1.57	92.6±5.5	94.1±2.4	90.7±5.6	95.2±3.7	93.9±2.8	95.3±3.1	93.7±2.2	-
0.79	92.3±5	94.0±2.2	93.9±5.0	96.7±2.1	95.6±3.2	94.4±2.9	92.7±1.6	-
0.4	92.6±5	95.4±2.6	98.5±1.9	95.2±2.7	94.4±2.3	94.5±3.1	95.9±3.1	-
0.2	94.6±4.2	95.3±1.6	96.7±2.4	95.5±2.1	93.9±6.7	94.3±3.2	92.9±3.1	-
0.1	95.2±4.5	93.1±2.3	94.9±4.2	95.3±3.4	94.3±2.3	95.2±3.8	96.1±3.6	-
-	-	-	-	-	-	-	-	92.9±8.8

Cells were treated for 24 h with peptides at a range of concentrations. The control corresponds to untreated cells (Control). The values are represented as the mean ± SD (n=8).

Table S3. Cytotoxic activity of melittin mutants against Expi293F cells.

Concentration (mkM)/ peptide	melittin	MR1P4	MR1P6	MR1P7	MR1P8	MR1P9	Control
200	1.4±0.6	2.2±0.9	0.5±0.5	0.0±0.0	2.6±0.8	0.6±0.7	-
100	1.0±0.7	1.4±1.6	0.3±0.6	0.6±0.6	1.9±0.9	6.0±4.8	-
50	1.1±0.5	1.0±1.3	1.8±0.8	0.7±0.7	5.9±4.5	35.8±12.2	-
25	4.0±1.9	2.3±3.3	15.3±11.5	0.0±0.0	35.4±15.2	72.6±4.9	-
12.5	8.6±4.3	4.8±8.0	37.3±9.3	1.5±2.1	79.8±9.4	80.7±8.3	-
6.25	13.6±7.1	15.7±13.3	69.2±8.7	22.5±13.3	89.0±6.1	89.5±6.1	-
3.13	23.9±4.7	31.3±9.4	88.6±7.2	57.4±12.4	93.3±5.5	94.6±3.1	-
1.57	42.8±9.4	49.4±11.2	94.7±3.3	91.6±3.8	94.1±4.6	95.5±3	-
0.79	61.7±7.8	76.8±12	95.7±3.7	95.4±3.0	94.3±5.5	96.8±2.1	-
0.4	79.7±6.2	87.2±7.9	94.4±2.6	95.0±3.5	92.3±7.5	93.5±1.8	-
0.2	92.4±4.2	90.9±7.7	94.5±2.5	93.5±3.4	94.8±3.6	93.2±3.2	-
-	-	-	-	-	-	-	88.9±7.8

Cells were treated for 24 h with peptides at a range of concentrations. The control corresponds to untreated cells (Control). The values are represented as the mean ± SD (n=8).

Table S4. Cytotoxic activity of Hm-AMP2 mutant peptides against Expi293F cells.

Concentration (mkM)/peptide	Hm-AMP2	A2R1P5	A2R2P3	A2R2P6	A2R2P7	A2R3P6	A2R3P7	A2R3P8	A2R3P10	A2R3P11	Control
200	38.3±8.5	69.4±10.1	73.1±9.5	65.1±12.8	10.5±3.9	80.2±13.1	43.1±14.7	23.8±11.3	79.8±9.5	55.7±12.6	-
100	87.3±11.0	94.0±6.0	91.0±3.5	91.8±6.2	54.8±21.2	93.8±4.5	73.8±10.3	36.0±9.2	94.2±4.1	61.9±10.7	-
50	93.1±5.7	93.8±3.9	92.5±4.1	93.2±6.2	85.1±10.5	94.2±3.2	93.7±7.3	53.2±9.7	95.9±2.8	75.9±10.9	-
25	92.8±4.3	93.6±4.1	94.5±3.7	94.6±6.1	93.0±6.5	97.0±1.4	95.4±2.6	75.7±7.7	96.1±4.5	95.6±3.2	-
12.5	95.9±5.4	94.8±3.6	95.0±3.1	93.4±3.2	93.6±3.5	96.9±2.5	96.2±2.8	91.1±6.3	95.4±3.6	94.2±3.2	-
6.25	92.2±7.5	95.0±3.8	94.7±2.0	95.1±3.2	94.8±3.1	96.1±2.5	92.9±2.0	95.3±2.5	96.0±2.7	95.3±2.8	-
3.13	92.7±5.8	92.4±4.4	94.5±3.4	95.2±3.2	94.6±5.1	94.0±2.5	93.7±3.0	94.6±3.3	96.8±1.9	92.0±3.5	-
1.57	91.5±3.8	89.6±15.6	95.6±2.9	93.7±2.5	95.9±2.5	96.8±1.8	94.4±2.0	95.6±2.2	98.0±3.3	94.6±3.1	-
0.79	95.9±1.9	94.9±3.5	95.9±3.6	94.0±2.7	94.3±3.3	95.7±2.1	95.0±2.8	94.8±2.8	96.1±3.0	95.9±1.6	-
0.4	94.3±4.5	95.6±3.9	95.9±2.0	94.2±5.5	93.5±5.7	94.6±1.9	94.3±2.4	95.3±1.9	96.0±1.6	95.5±1.7	-
0.2	94.8±3.3	91.6±5.6	94.7±2.2	90.8±6.9	94.6±6.4	94.8±2.6	93.7±5.2	96.3±2.4	95.5±2.5	96.2±3.2	-
0.1	-	-	-	-	-	-	-	-	-	-	95.2±2.4

Cells were treated for 24 h with peptides at a range of concentrations. The control corresponds to untreated cells (Control). The values are represented as the mean ± SD (n=8).

Figures

A

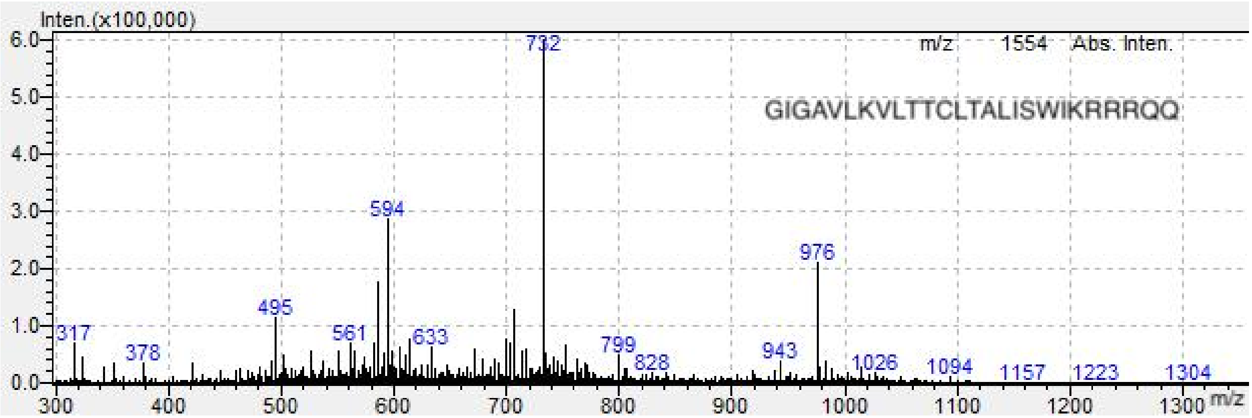

B

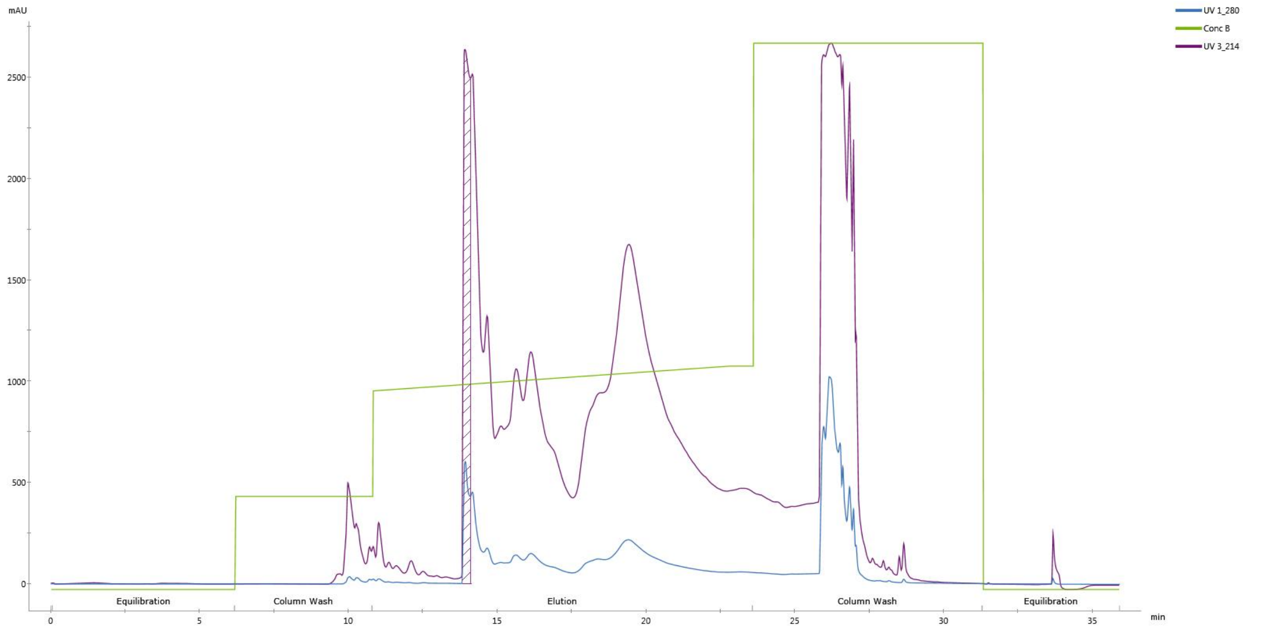

C

Peptide name	MR1P4
Peptide sequence	GIGAVLKVLTTCLTALISWIKRRRQQ
Molecular weight	2926
Resin type	Fmoc-Rink amide aminomethyl-polystyrene resin (ABCR GmbH & Co KG, Karlsruhe, Germany); loading capacity 0.78 mmol/g

Figure S1. Datasheet of peptide MR1P4. A - Mass spectrum of peptide MR1P4. B - Chromatogram of preparative HPLC purification of peptide MR1P4. Chromatogram showing the gradient profile (green), UV absorbance at 280 nm (blue) and 214 nm (violet), with the target peak indicated by the shaded violet region. C- Characterization of peptide MR1P4.

A

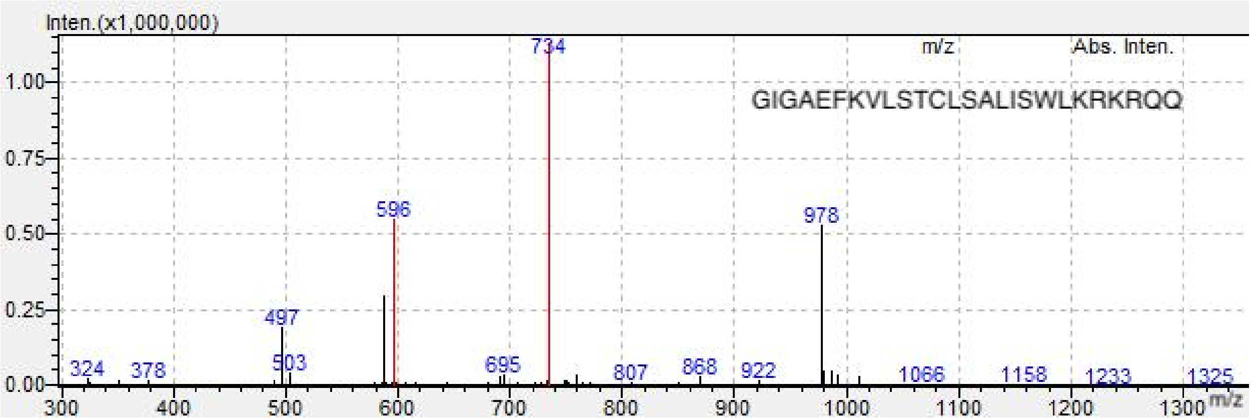

B

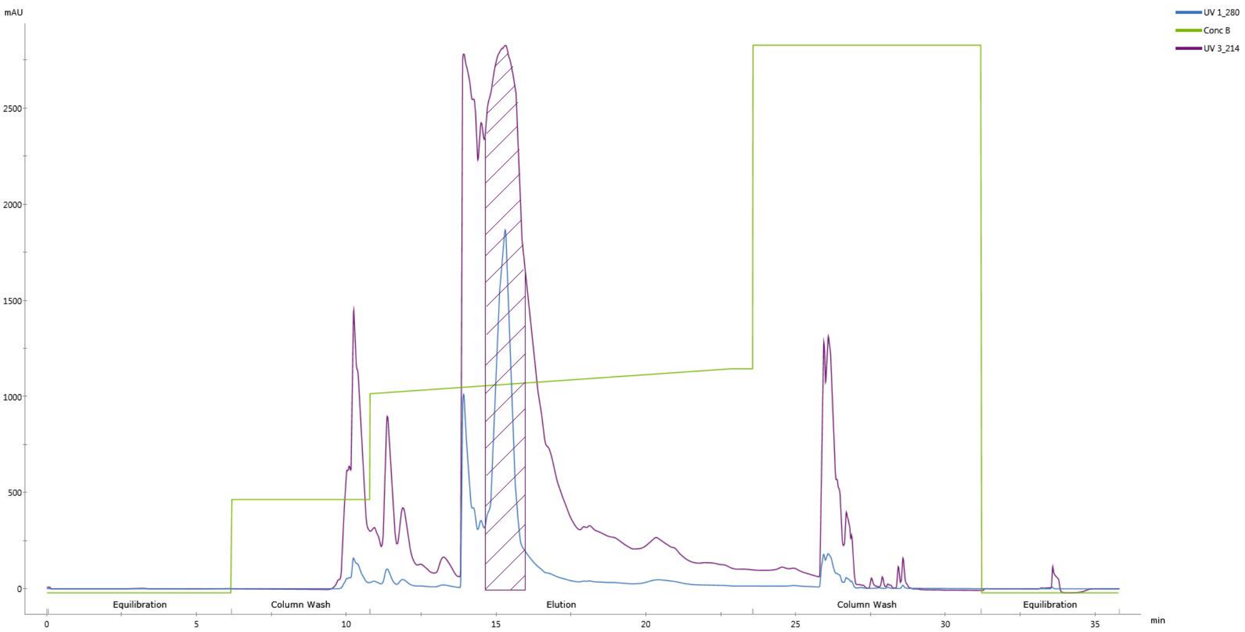

C

Peptide name	MR1P6
Peptide sequence	GIGAEFKVLSTCLSALISWLKRKRQQ
Molecular weight	2934
Resin type	Fmoc-Rink amide aminomethyl-polystyrene resin (ABCR GmbH & Co KG, Karlsruhe, Germany); loading capacity 0.78 mmol/g

Figure S2. Datasheet of peptide MR1P6. A - Mass spectrum of peptide MR1P6. B - Chromatogram of preparative HPLC purification of peptide MR1P6. Chromatogram showing the gradient profile (green), UV absorbance at 280 nm (blue) and 214 nm (violet), with the target peak indicated by the shaded violet region. C- Characterization of peptide MR1P6.

A

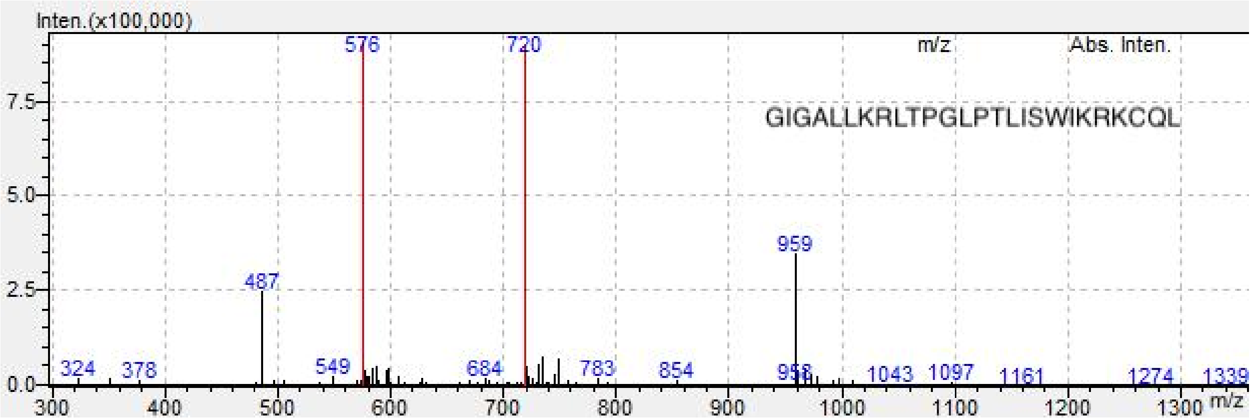

B

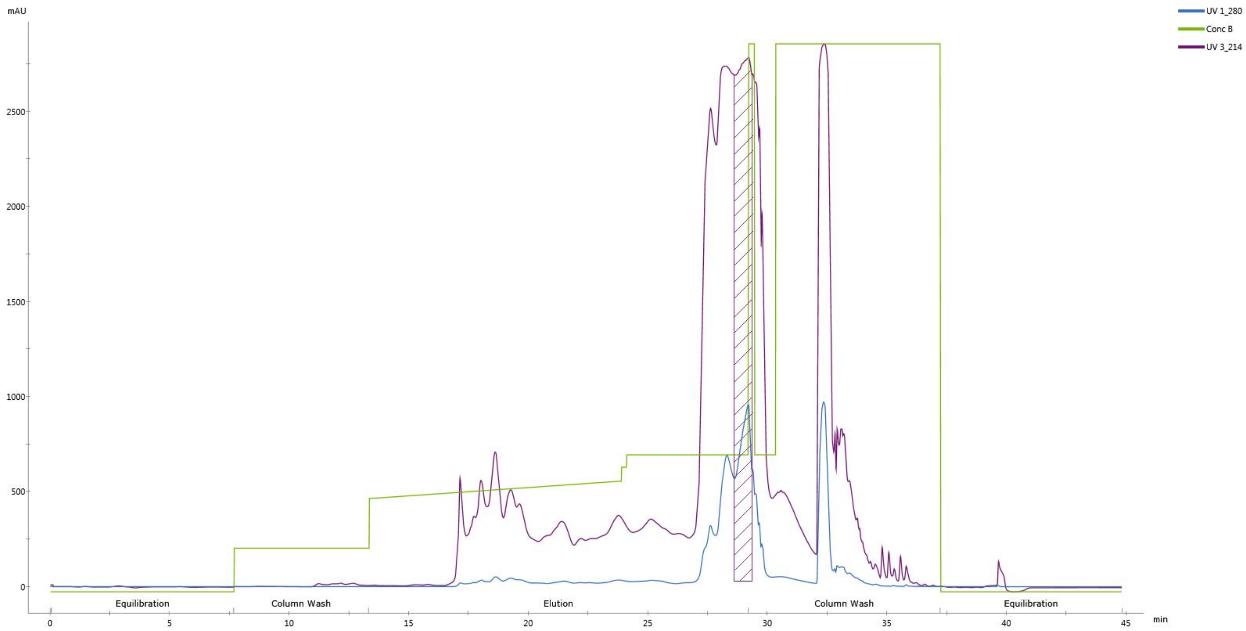

C

Peptide name	MR1P7
Peptide sequence	GIGALLKRLTPGLPTLISWIKRKCQL
Molecular weight	2877
Resin type	Fmoc-Rink amide aminomethyl-polystyrene resin (ABCR GmbH & Co KG, Karlsruhe, Germany); loading capacity 0.78 mmol/g

Figure S3. Datasheet of peptide MR1P7. A - Mass spectrum of peptide MR1P7. B - Chromatogram of preparative HPLC purification of peptide MR1P7. Chromatogram showing the gradient profile (green), UV absorbance at 280 nm (blue) and 214 nm (violet), with the target peak indicated by the shaded violet region. C- Characterization of peptide MR1P7.

A

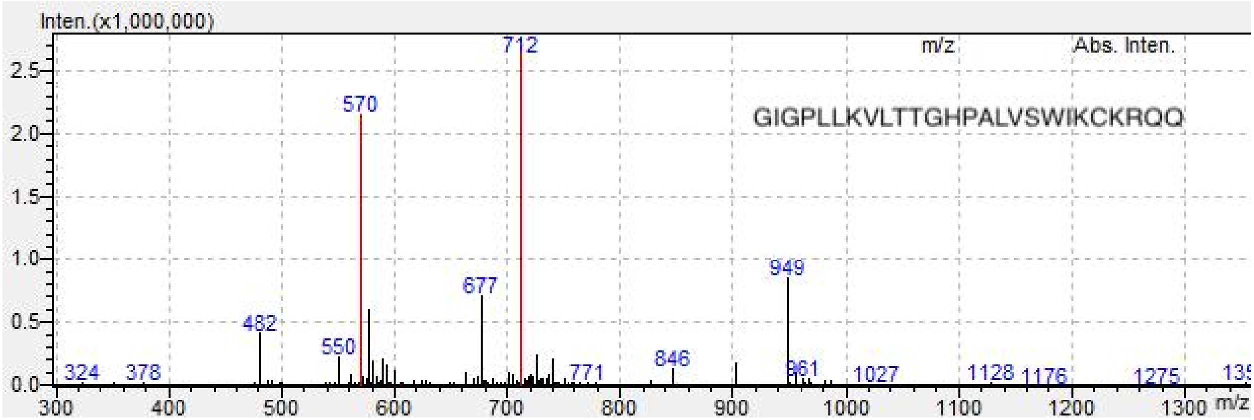

B

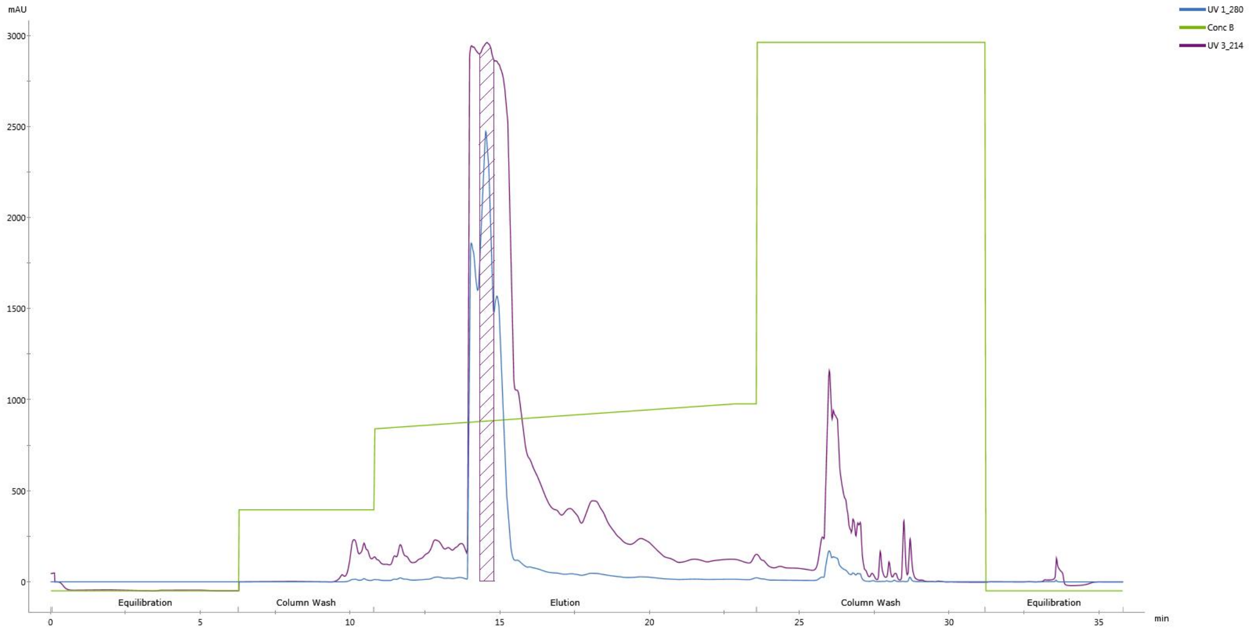

C

Peptide name	MR1P8
Peptide sequence	GIGPLLKVLTTGHPALVSWIKCKRQQ
Molecular weight	2844
Resin type	Fmoc-Rink amide aminomethyl-polystyrene resin (ABCR GmbH & Co KG, Karlsruhe, Germany); loading capacity 0.78 mmol/g

Figure S4. Datasheet of peptide MR1P8. A - Mass spectrum of peptide MR1P8. B - Chromatogram of preparative HPLC purification of peptide MR1P8. Chromatogram showing the gradient profile (green), UV absorbance at 280 nm (blue) and 214 nm (violet), with the target peak indicated by the shaded violet region. C- Characterization of peptide MR1P8.

A

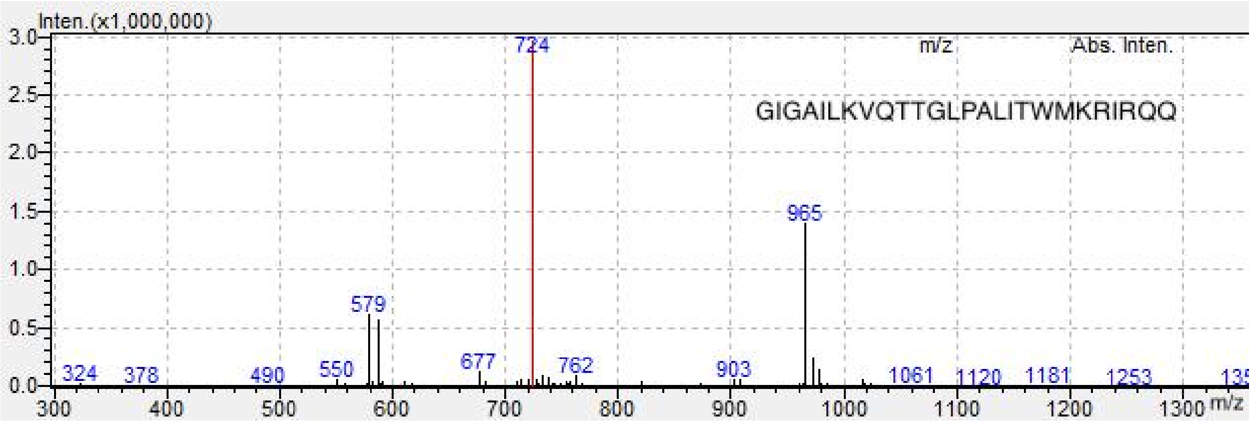

B

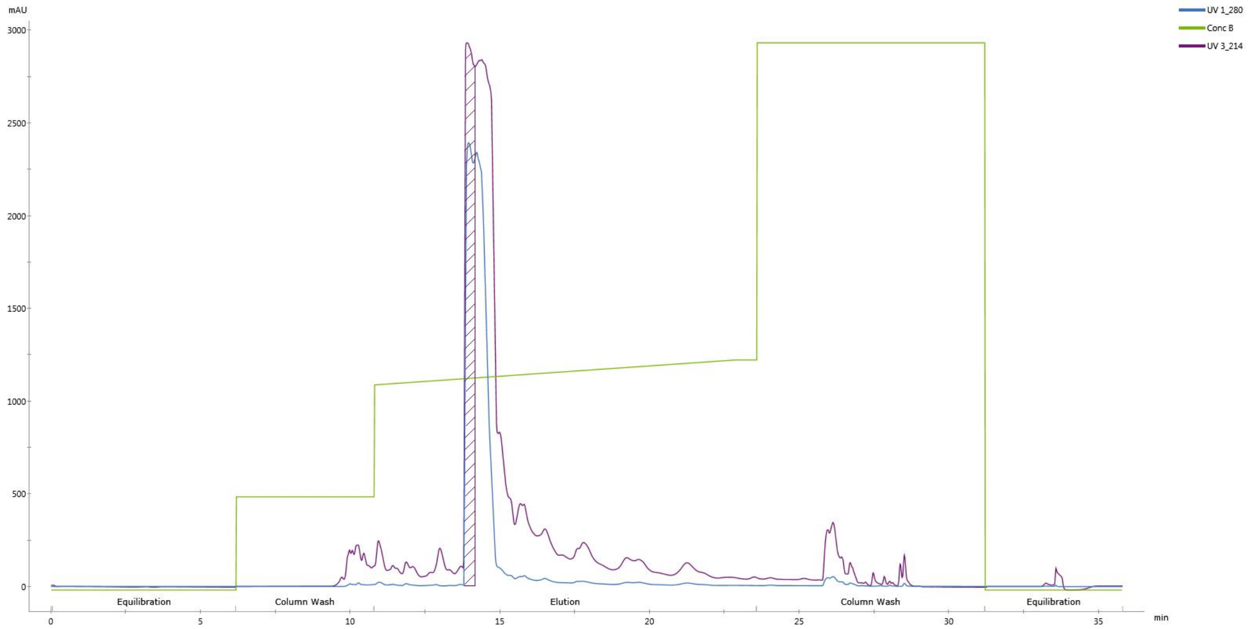

C

Peptide name	MR1P9
Peptide sequence	GIGAILKVQTTGLPALITWMKRIRQQ
Molecular weight	2894
Resin type	Fmoc-Rink amide aminomethyl-polystyrene resin (ABCR GmbH & Co KG, Karlsruhe, Germany); loading capacity 0.78 mmol/g

Figure S5. Datasheet of peptide MR1P9. A - Mass spectrum of peptide MR1P9. B - Chromatogram of preparative HPLC purification of peptide MR1P9. Chromatogram showing the gradient profile (green), UV absorbance at 280 nm (blue) and 214 nm (violet), with the target peak indicated by the shaded violet region. C- Characterization of peptide MR1P9.

A

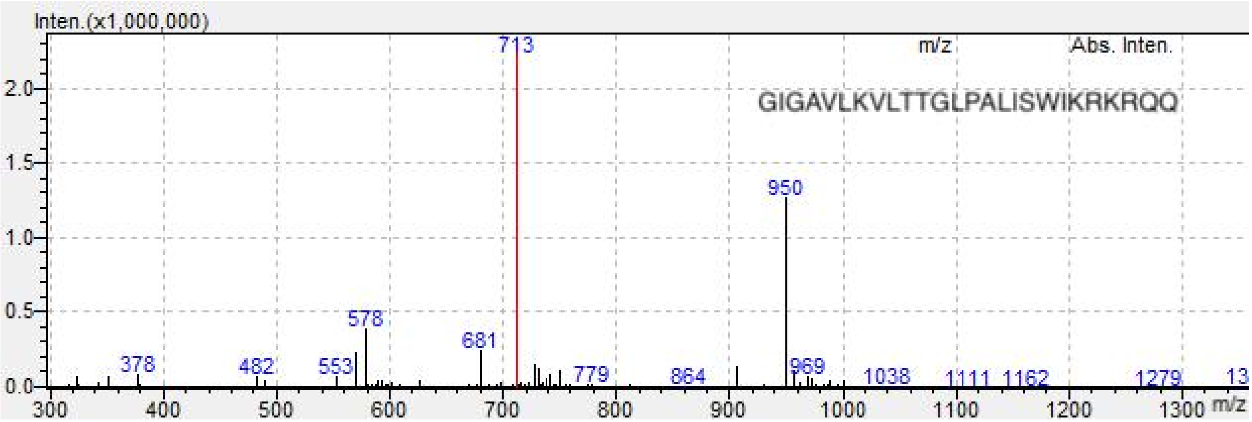

B

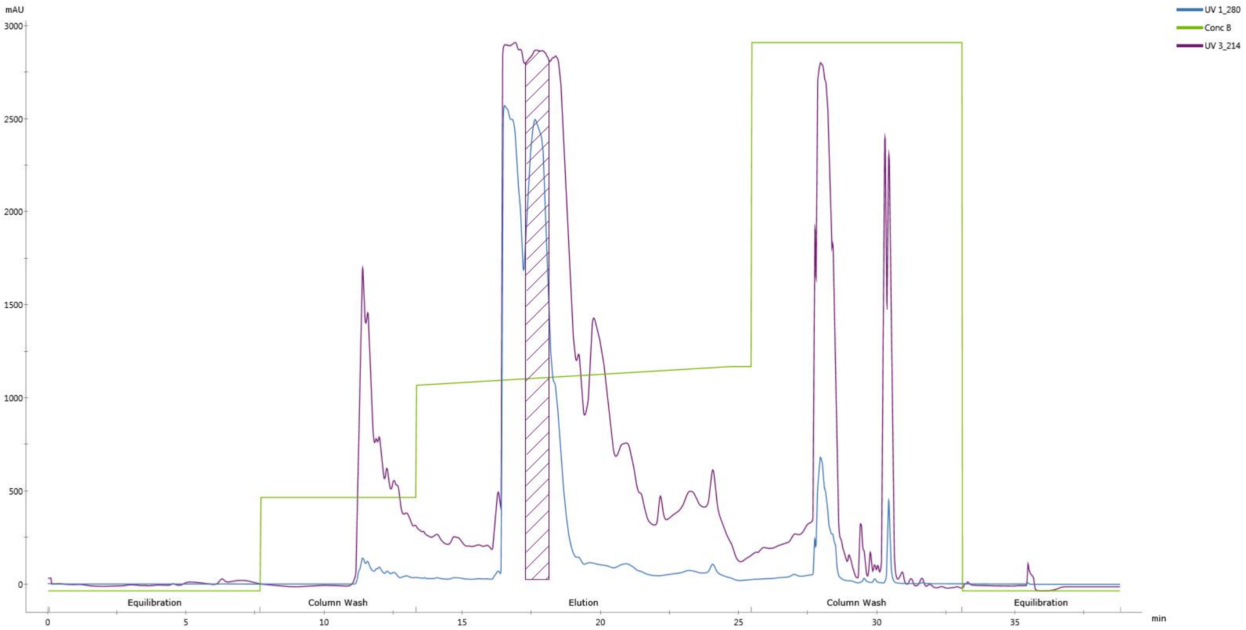

C

Peptide name	melittin
Peptide sequence	GIGAVLKVLTTGLPALISWIKRKRQQ
Molecular weight	2848
Resin type	Fmoc-Rink amide aminomethyl-polystyrene resin (ABCR GmbH & Co KG, Karlsruhe, Germany); loading capacity 0.78 mmol/g

Figure S6. Datasheet of peptide melittin. A - Mass spectrum of peptide melittin. B - Chromatogram of preparative HPLC purification of peptide melittin. Chromatogram showing the gradient profile (green), UV absorbance at 280 nm (blue) and 214 nm (violet), with the target peak indicated by the shaded violet region. C- Characterization of peptide melittin.

A

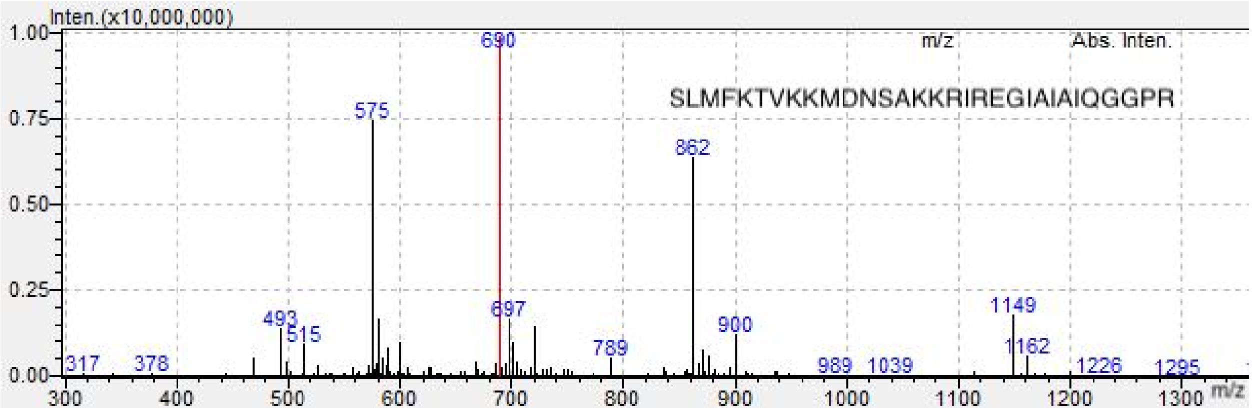

B

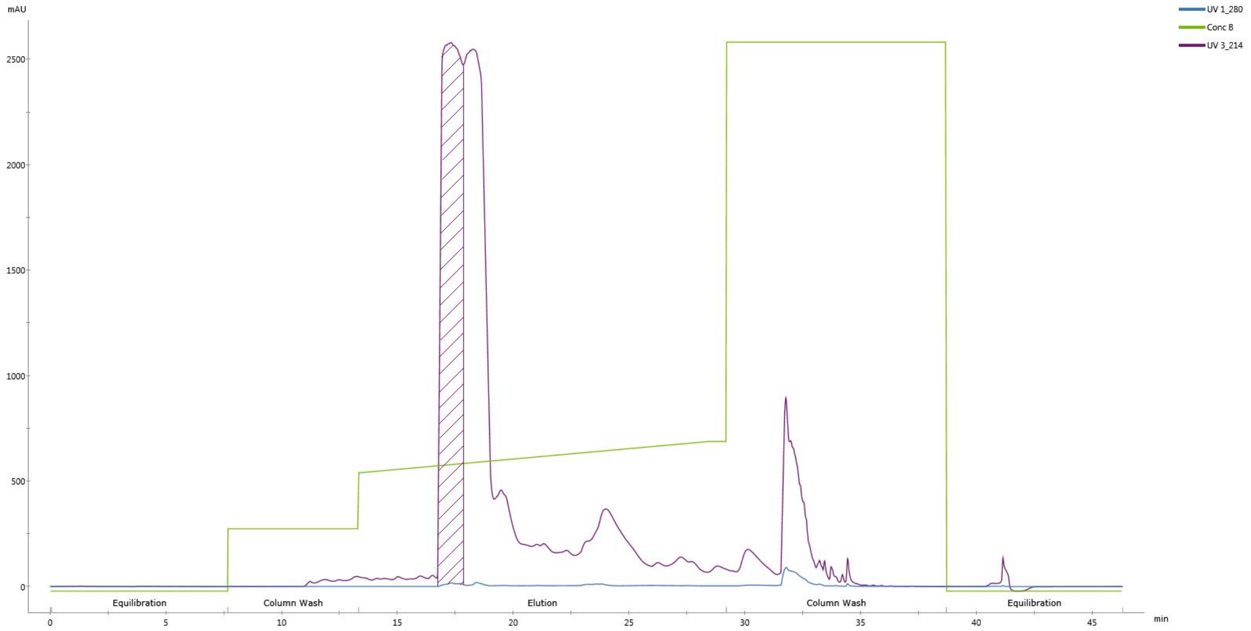

C

Peptide name	CR1P5
Peptide sequence	SLMFKTVKKMDNSAKKRIREGIAIAIQGGPR
Molecular weight	3445
Resin type	Fmoc-Rink amide PEG MBHA resin (ABCR GmbH & Co KG, Karlsruhe, Germany); loading capacity 0.48 mmol/g

Figure S7. Datasheet of peptide CR1P5. A - Mass spectrum of peptide CR1P5. B - Chromatogram of preparative HPLC purification of peptide CR1P5. Chromatogram showing the gradient profile (green), UV absorbance at 280 nm (blue) and 214 nm (violet), with the target peak indicated by the shaded violet region. C- Characterization of peptide CR1P5.

A

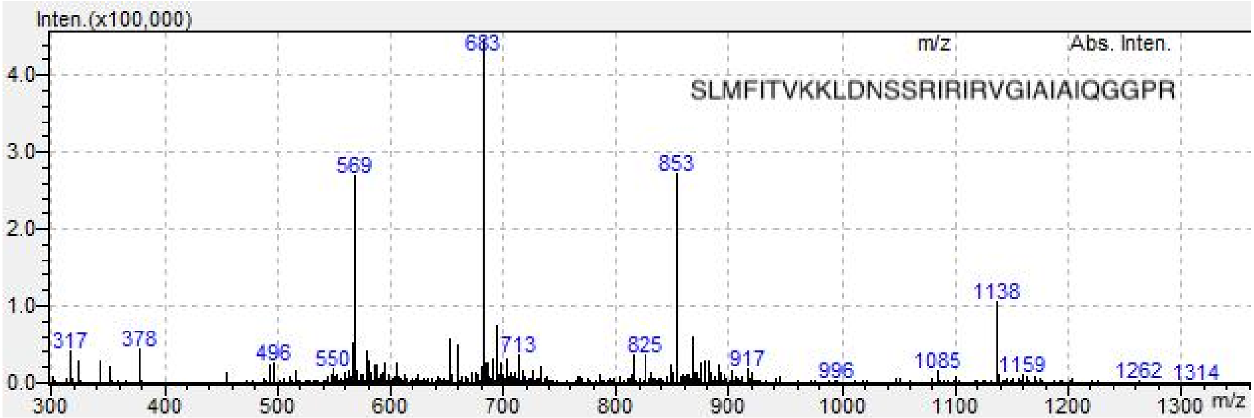

B

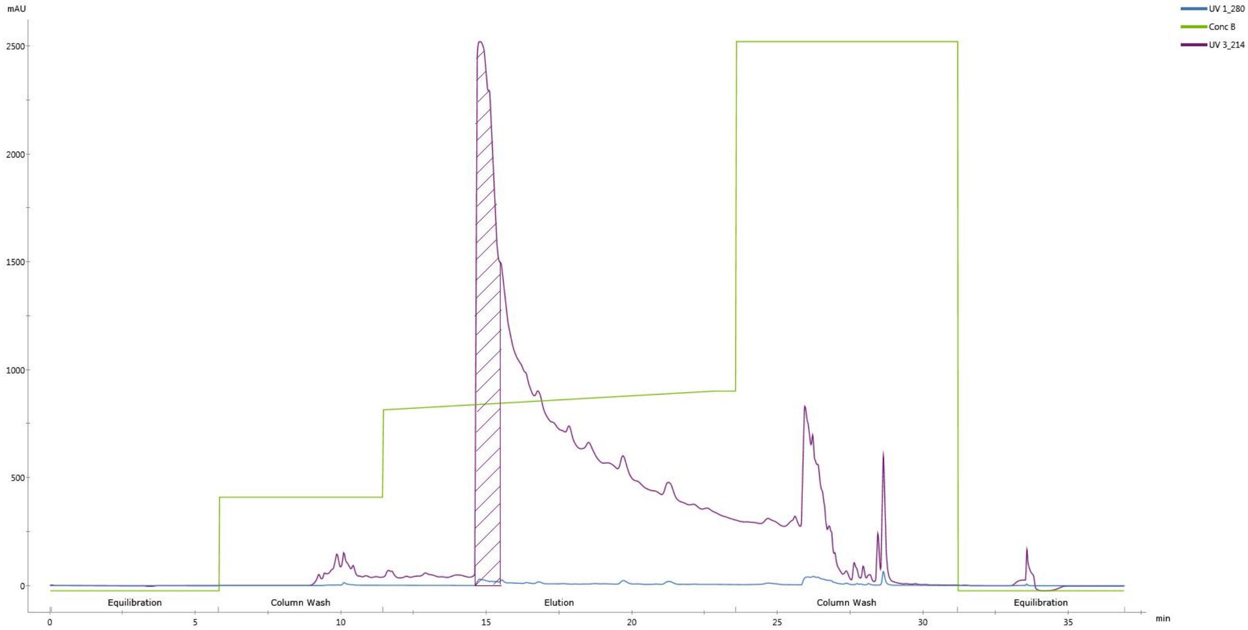

C

Peptide name	CR2P2
Peptide sequence	SLMFITVKKLDNSSRIRIRVGIAIAIQGGPR
Molecular weight	3411
Resin type	Fmoc-Rink amide PEG MBHA resin (ABCR GmbH & Co KG, Karlsruhe, Germany); loading capacity 0.48 mmol/g

Figure S8. Datasheet of peptide CR2P2. A - Mass spectrum of peptide CR2P2. B - Chromatogram of preparative HPLC purification of peptide CR2P2. Chromatogram showing the gradient profile (green), UV absorbance at 280 nm (blue) and 214 nm (violet), with the target peak indicated by the shaded violet region. C- Characterization of peptide CR2P2.

A

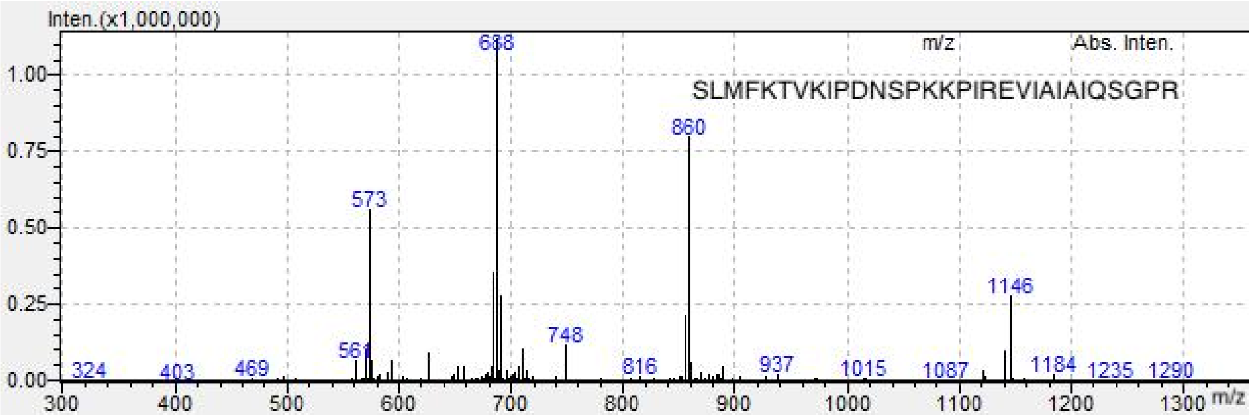

B

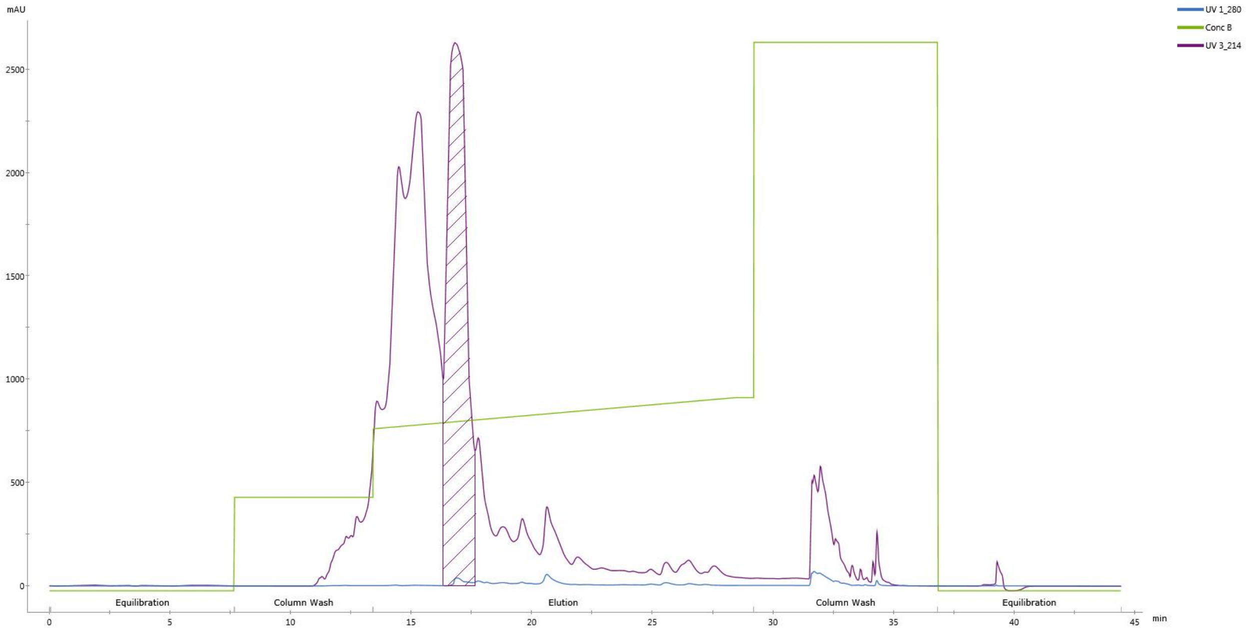

C

Peptide name	CR2P6
Peptide sequence	SLMFKTVKIPDNSPKKPIREVIAIAIQSGPR
Molecular weight	3435
Resin type	Fmoc-Rink amide PEG MBHA resin (ABCR GmbH & Co KG, Karlsruhe, Germany); loading capacity 0.48 mmol/g

Figure S9. Datasheet of peptide CR2P6. A - Mass spectrum of peptide CR2P6. B - Chromatogram of preparative HPLC purification of peptide CR2P6. Chromatogram showing the gradient profile (green), UV absorbance at 280 nm (blue) and 214 nm (violet), with the target peak indicated by the shaded violet region. C- Characterization of peptide CR2P6.

A

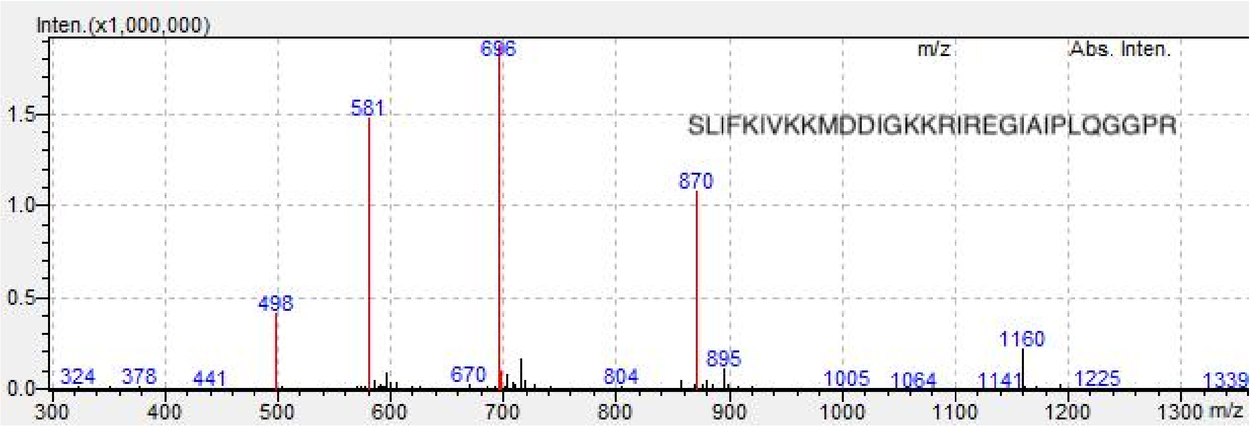

B

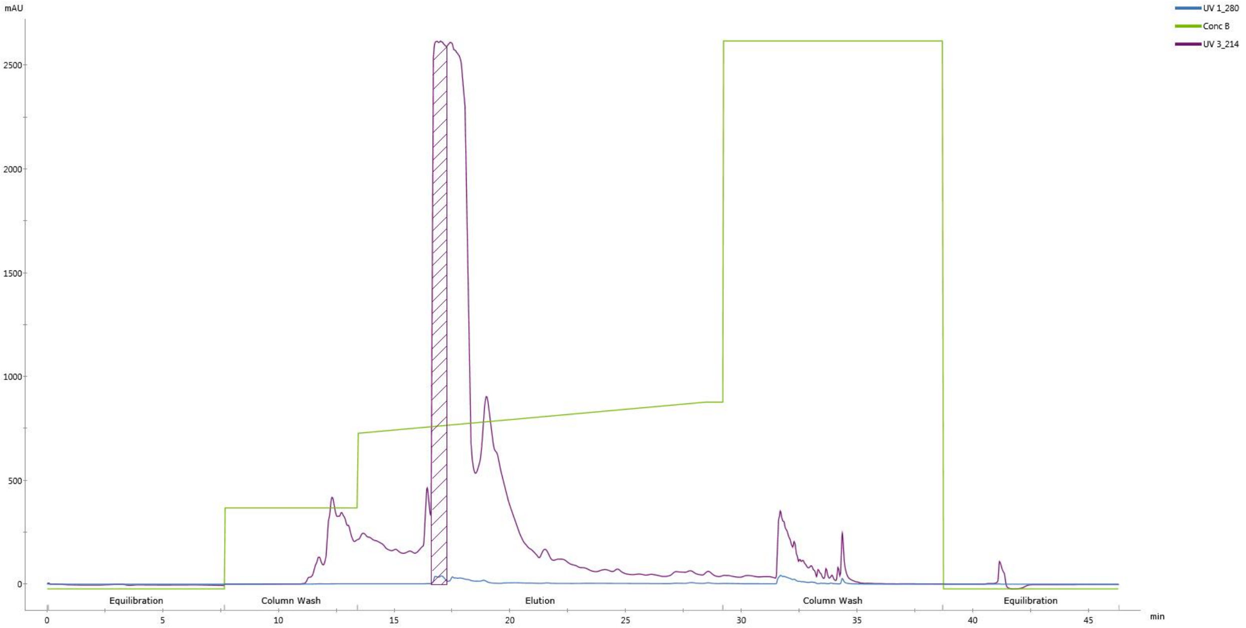

C

Peptide name	CR2P7
Peptide sequence	SLIFKIVKKMDDIGKKRIREGIAIPLQGGPR
Molecular weight	3478
Resin type	Fmoc-Rink amide PEG MBHA resin (ABCR GmbH & Co KG, Karlsruhe, Germany); loading capacity 0.48 mmol/g

Figure S10. Datasheet of peptide CR2P7. A - Mass spectrum of peptide CR2P7. B - Chromatogram of preparative HPLC purification of peptide CR2P7. Chromatogram showing the gradient profile (green), UV absorbance at 280 nm (blue) and 214 nm (violet), with the target peak indicated by the shaded violet region. C- Characterization of peptide CR2P7.

A

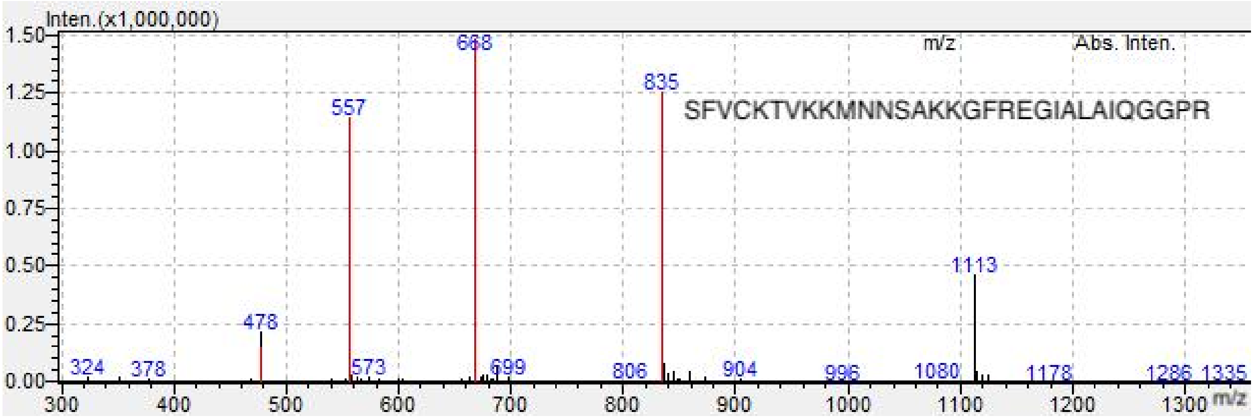

B

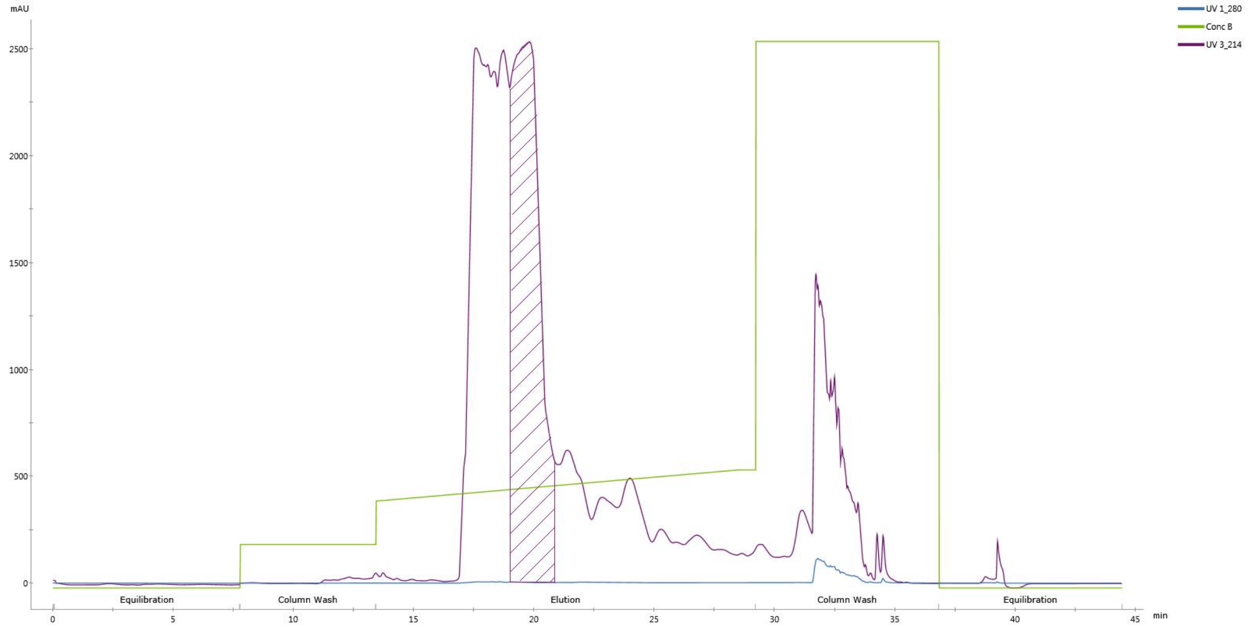

C

Peptide name	CR2P8
Peptide sequence	SFVCKTVKKMNNSAKKGFREGIALAIQGGPR
Molecular weight	3337
Resin type	Fmoc-Rink amide PEG MBHA resin (ABCR GmbH & Co KG, Karlsruhe, Germany); loading capacity 0.48 mmol/g

Figure S11. Datasheet of peptide CR2P8. A - Mass spectrum of peptide CR2P8. B - Chromatogram of preparative HPLC purification of peptide CR2P8. Chromatogram showing the gradient profile (green), UV absorbance at 280 nm (blue) and 214 nm (violet), with the target peak indicated by the shaded violet region. C- Characterization of peptide CR2P8.

A

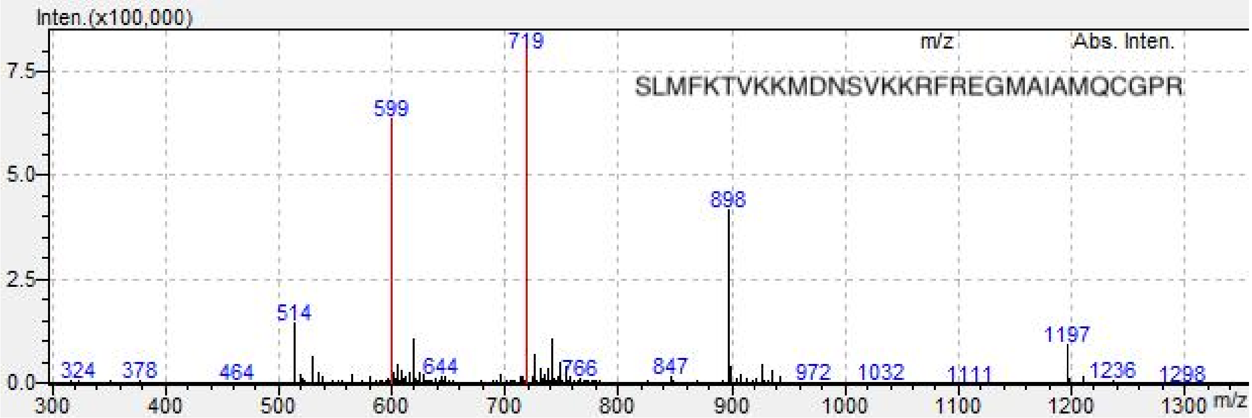

B

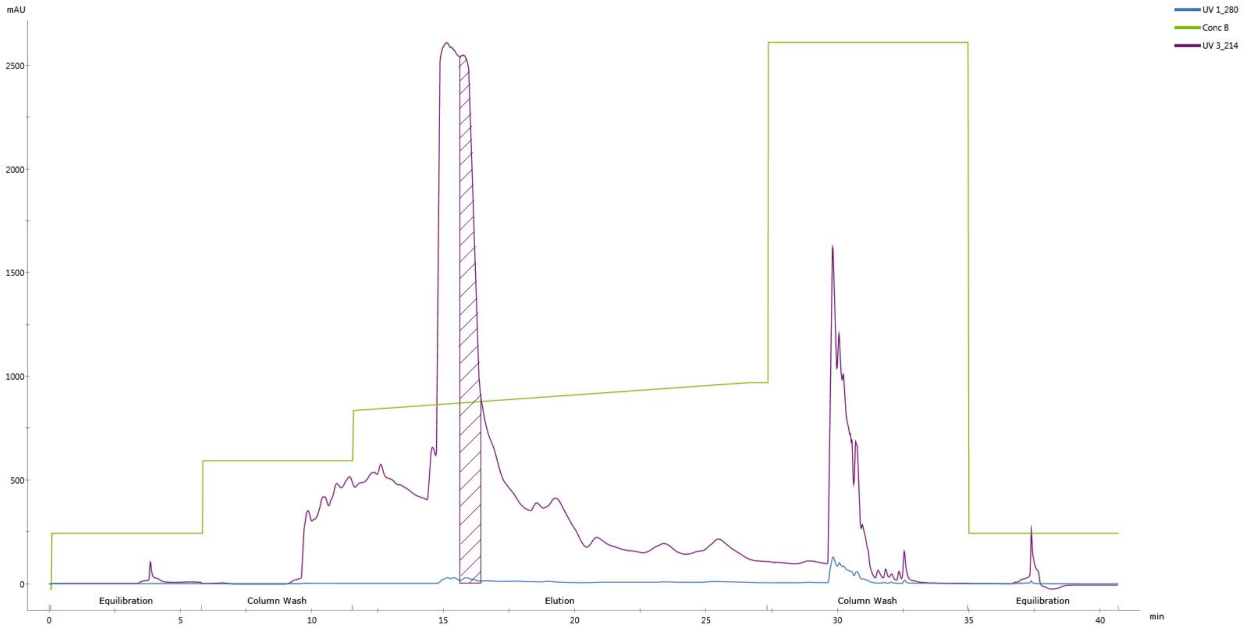

C

Peptide name	CR2P9
Peptide sequence	SLMFKTVKKMDNSVKKRFREGMAIAMQCGPR
Molecular weight	3589
Resin type	Fmoc-Rink amide PEG MBHA resin (ABCR GmbH & Co KG, Karlsruhe, Germany); loading capacity 0.48 mmol/g

Figure S12. Datasheet of peptide CR2P9. A - Mass spectrum of peptide CR2P9. B - Chromatogram of preparative HPLC purification of peptide CR2P9. Chromatogram showing the gradient profile (green), UV absorbance at 280 nm (blue) and 214 nm (violet), with the target peak indicated by the shaded violet region. C- Characterization of peptide CR2P9.

A

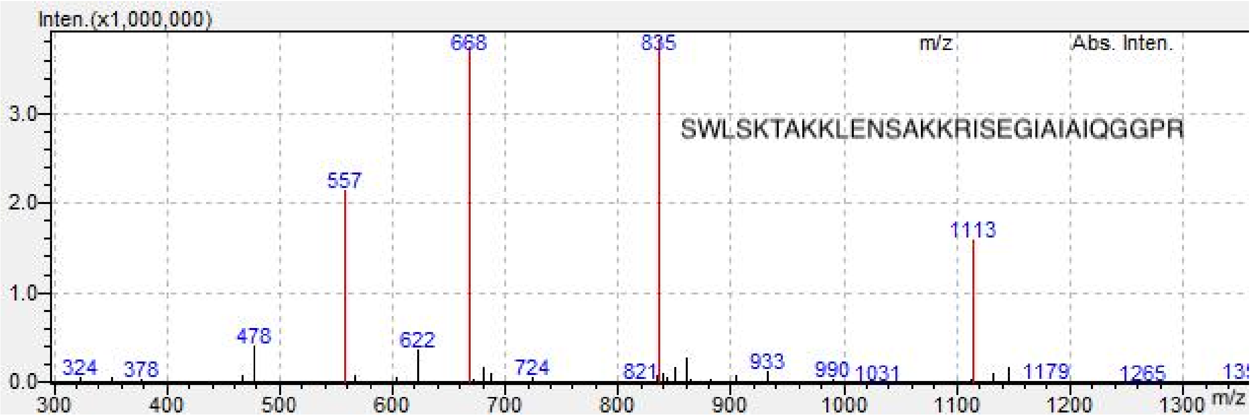

B

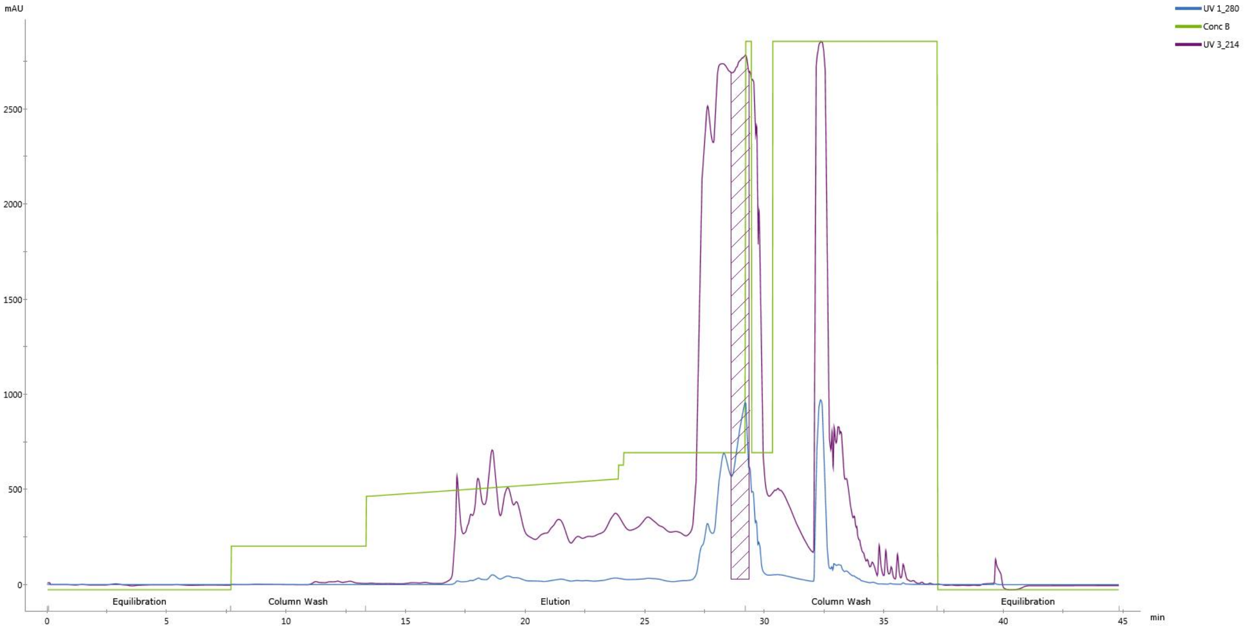

C

Peptide name	cecropin
Peptide sequence	SWLSKTAKKLENSAKKRISEGIAIAIQGGPR
Molecular weight	3339
Resin type	Fmoc-Rink amide PEG MBHA resin (ABCR GmbH & Co KG, Karlsruhe, Germany); loading capacity 0.48 mmol/g

Figure S13. Datasheet of peptide cecropin. A - Mass spectrum of peptide cecropin. B - Chromatogram of preparative HPLC purification of peptide cecropin. Chromatogram showing the gradient profile (green), UV absorbance at 280 nm (blue) and 214 nm (violet), with the target peak indicated by the shaded violet region. C- Characterization of peptide cecropin.

A

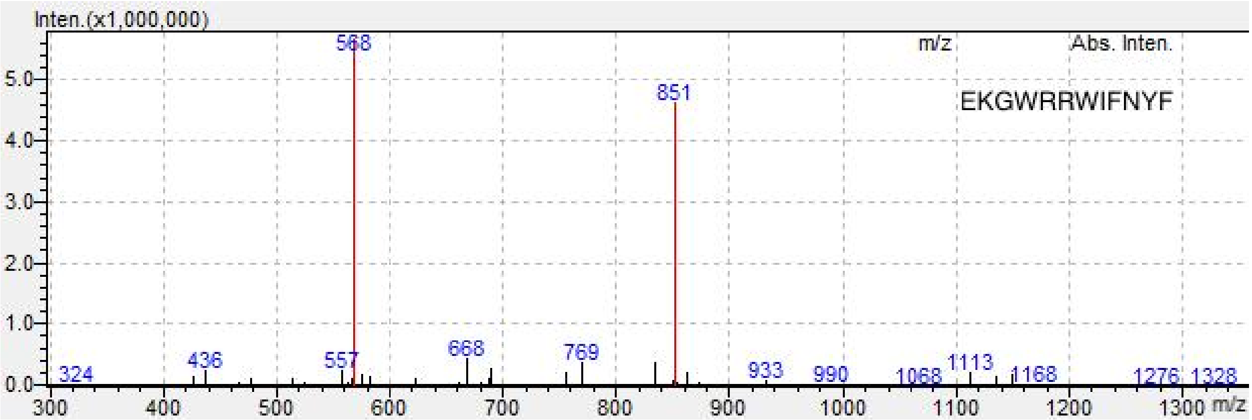

B

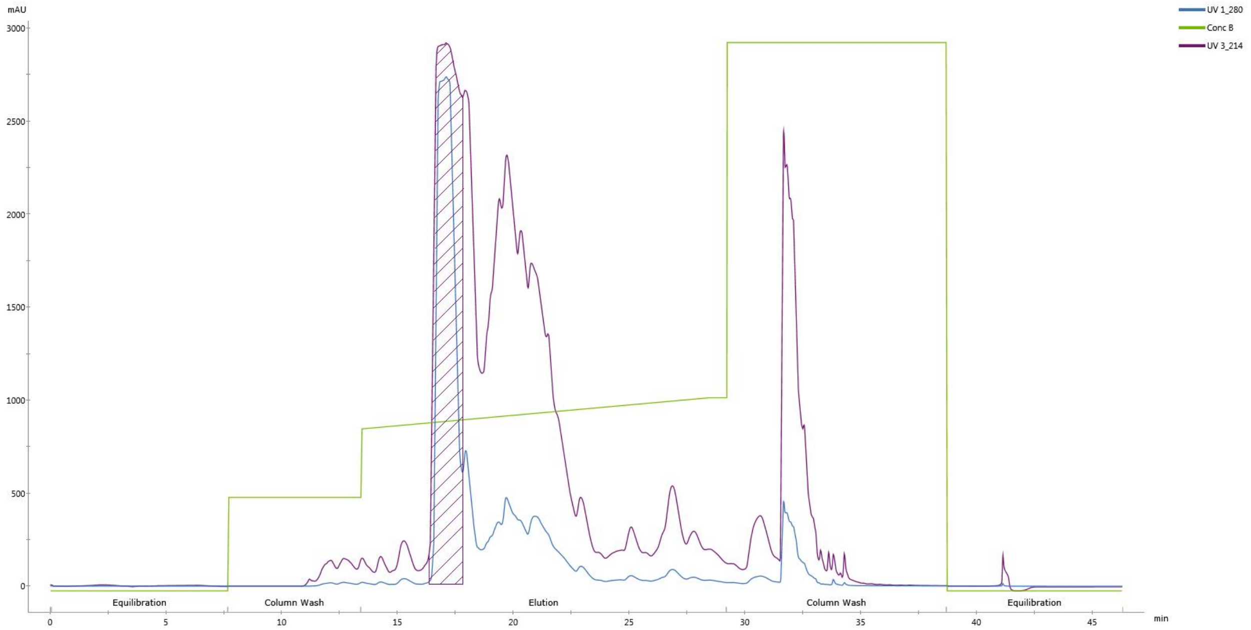

C

Peptide name	A2R1P5
Peptide sequence	EKGWRRWIFNYF
Molecular weight	1702
Resin type	Fmoc-Rink amide aminomethyl-polystyrene resin (ABCR GmbH & Co KG, Karlsruhe, Germany); loading capacity 0.78 mmol/g

Figure S14. Datasheet of peptide A2R1P5. A - Mass spectrum of peptide A2R1P5. B - Chromatogram of preparative HPLC purification of peptide A2R1P5. Chromatogram showing the gradient profile (green), UV absorbance at 280 nm (blue) and 214 nm (violet), with the target peak indicated by the shaded violet region. C- Characterization of peptide A2R1P5.

A

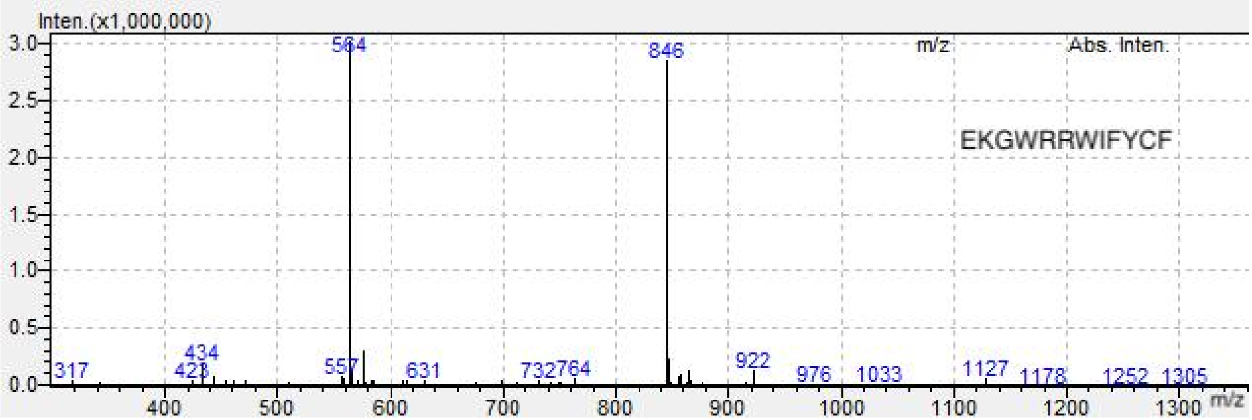

B

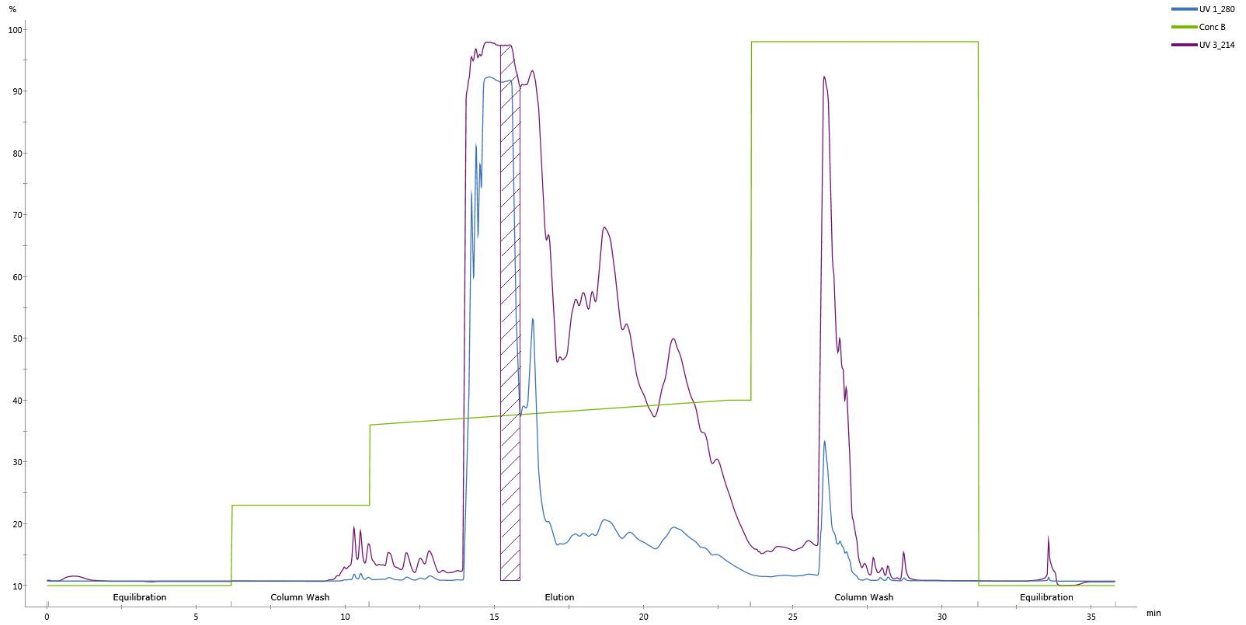

C

Peptide name	A2R2P3
Peptide sequence	EKGWRRWIFYCF
Molecular weight	1691
Resin type	Fmoc-Rink amide aminomethyl-polystyrene resin (ABCR GmbH & Co KG, Karlsruhe, Germany); loading capacity 0.78 mmol/g

Figure S15. Datasheet of peptide A2R2P3. A - Mass spectrum of peptide A2R2P3. B - Chromatogram of preparative HPLC purification of peptide A2R2P3. Chromatogram showing the gradient profile (green), UV absorbance at 280 nm (blue) and 214 nm (violet), with the target peak indicated by the shaded violet region. C- Characterization of peptide A2R2P3.

A

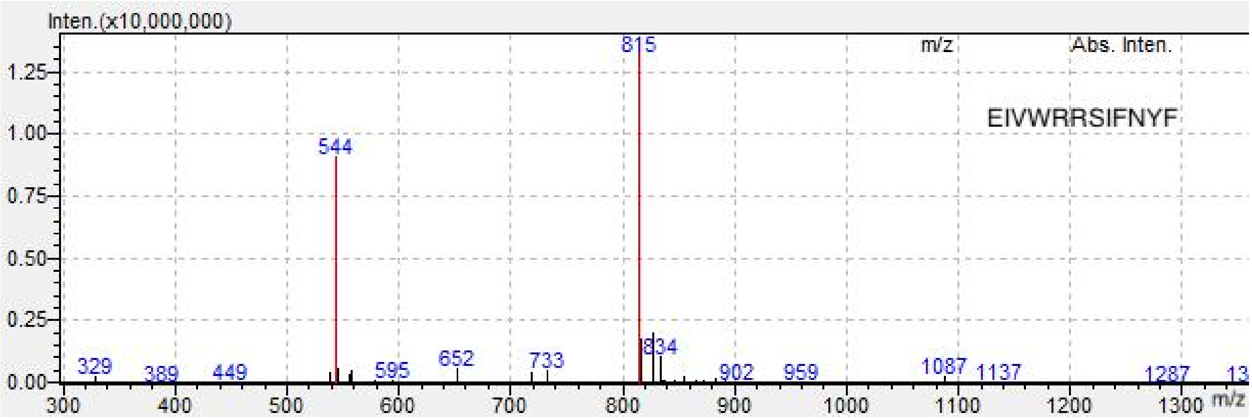

B

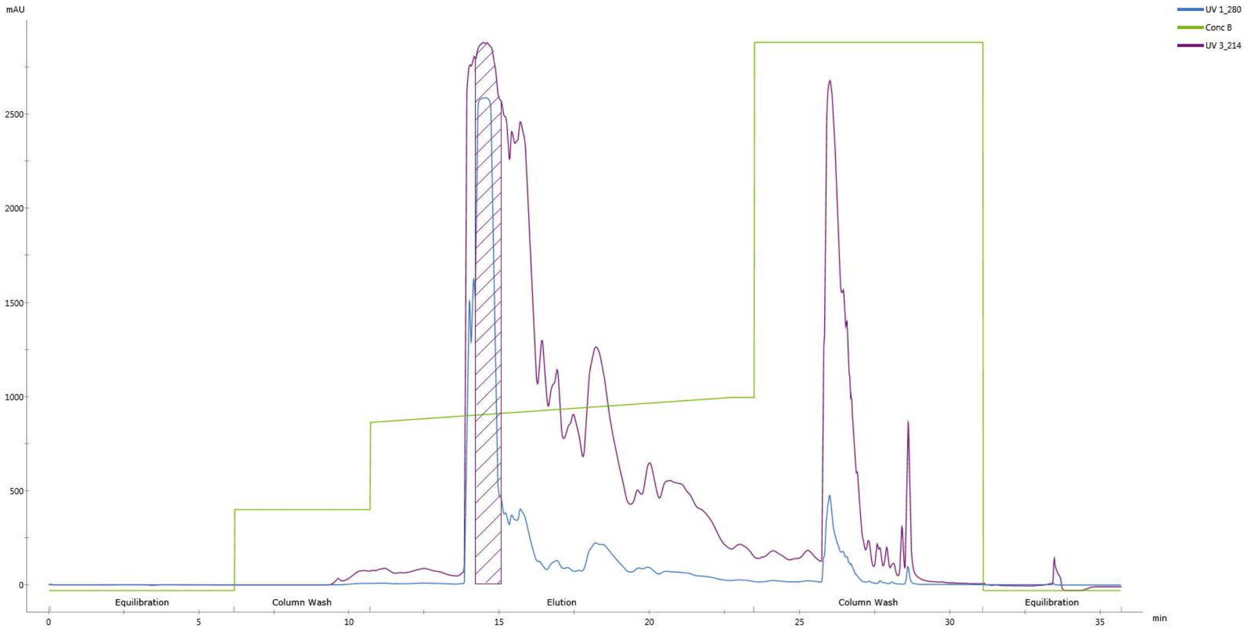

C

Peptide name	A2R2P6
Peptide sequence	EIVWRRSIFNYF
Molecular weight	1630
Resin type	Fmoc-Rink amide aminomethyl-polystyrene resin (ABCR GmbH & Co KG, Karlsruhe, Germany); loading capacity 0.78 mmol/g

Figure S16. Datasheet of peptide A2R2P6. A - Mass spectrum of peptide A2R2P6. B - Chromatogram of preparative HPLC purification of peptide A2R2P6. Chromatogram showing the gradient profile (green), UV absorbance at 280 nm (blue) and 214 nm (violet), with the target peak indicated by the shaded violet region. C- Characterization of peptide A2R2P6.

A

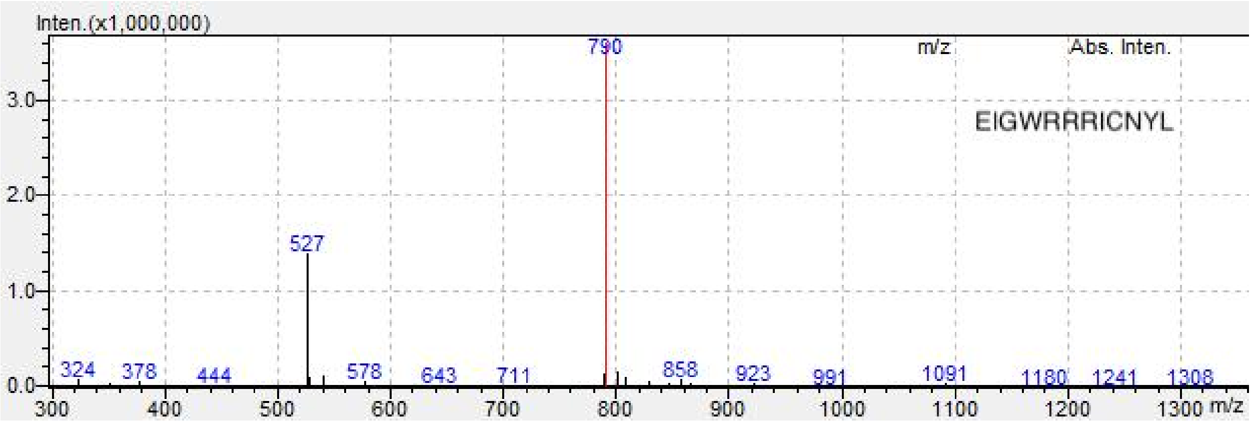

B

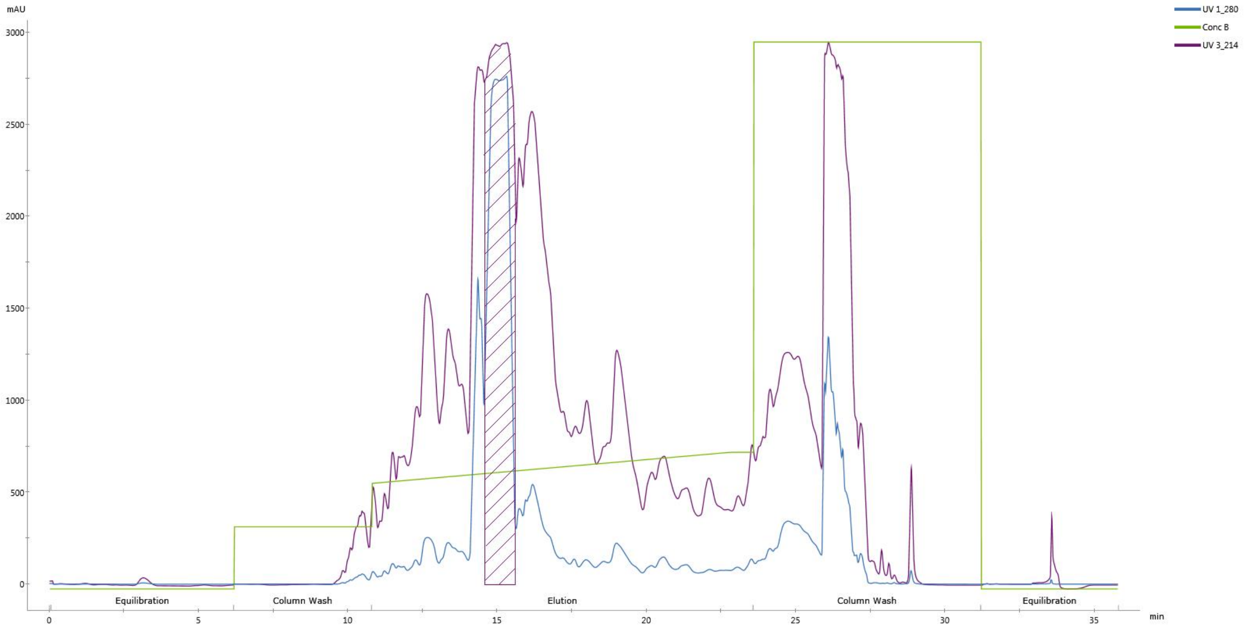

C

Peptide name	A2R2P7
Peptide sequence	EIGWRRRICNYL
Molecular weight	1579
Resin type	Fmoc-Rink amide aminomethyl-polystyrene resin (ABCR GmbH & Co KG, Karlsruhe, Germany); loading capacity 0.78 mmol/g

Figure S17. Datasheet of peptide A2R2P7. A - Mass spectrum of peptide A2R2P7. B - Chromatogram of preparative HPLC purification of peptide A2R2P7. Chromatogram showing the gradient profile (green), UV absorbance at 280 nm (blue) and 214 nm (violet), with the target peak indicated by the shaded violet region. C- Characterization of peptide A2R2P7.

A

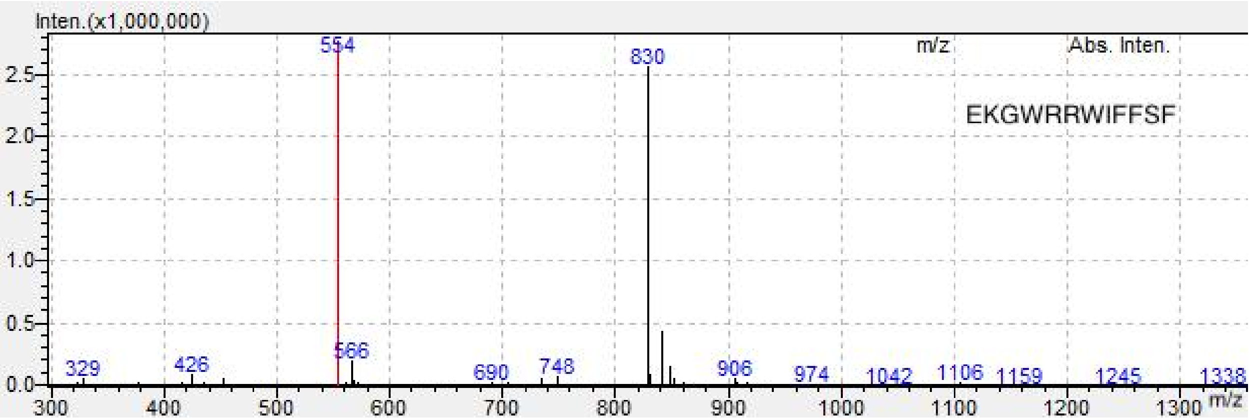

B

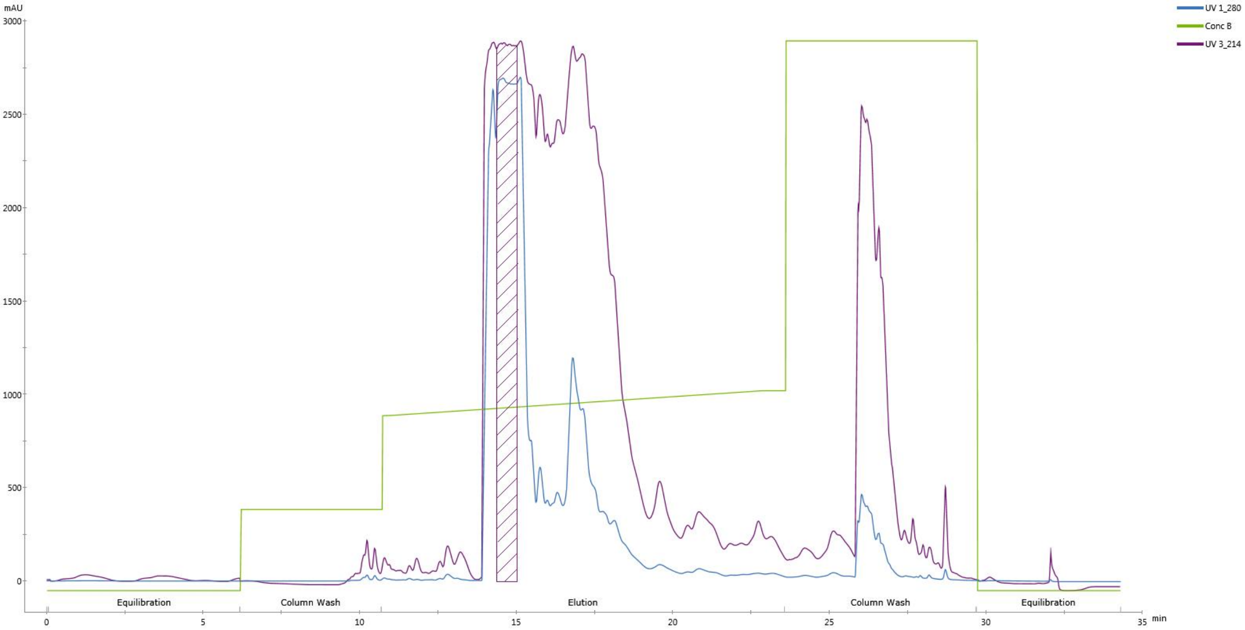

C

Peptide name	A2R3P6
Peptide sequence	EKGWRRWIFFSF
Molecular weight	1659
Resin type	Fmoc-Rink amide aminomethyl-polystyrene resin (ABCR GmbH & Co KG, Karlsruhe, Germany); loading capacity 0.78 mmol/g

Figure S18. Datasheet of peptide A2R3P6. A - Mass spectrum of peptide A2R3P6. B - Chromatogram of preparative HPLC purification of peptide A2R3P6. Chromatogram showing the gradient profile (green), UV absorbance at 280 nm (blue) and 214 nm (violet), with the target peak indicated by the shaded violet region. C- Characterization of peptide A2R3P6.

A

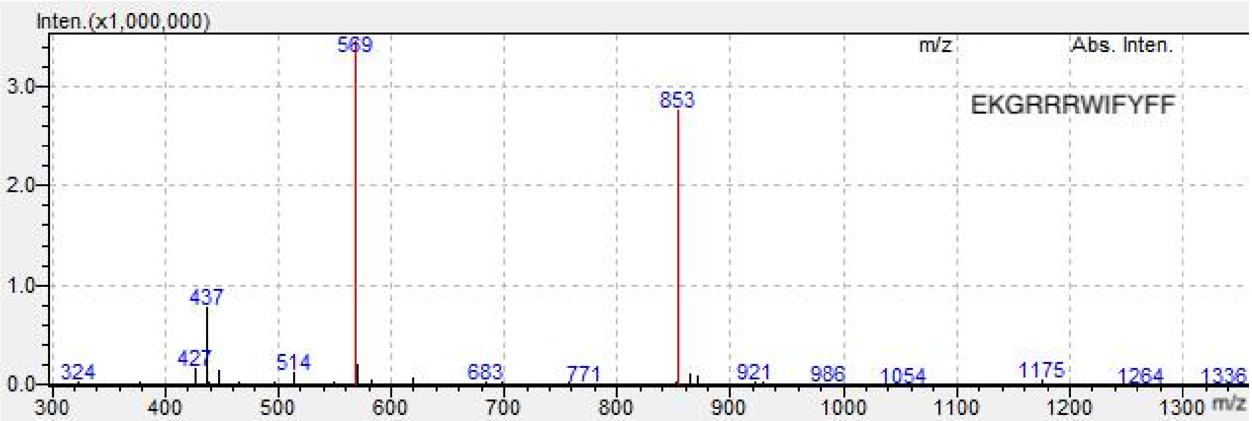

B

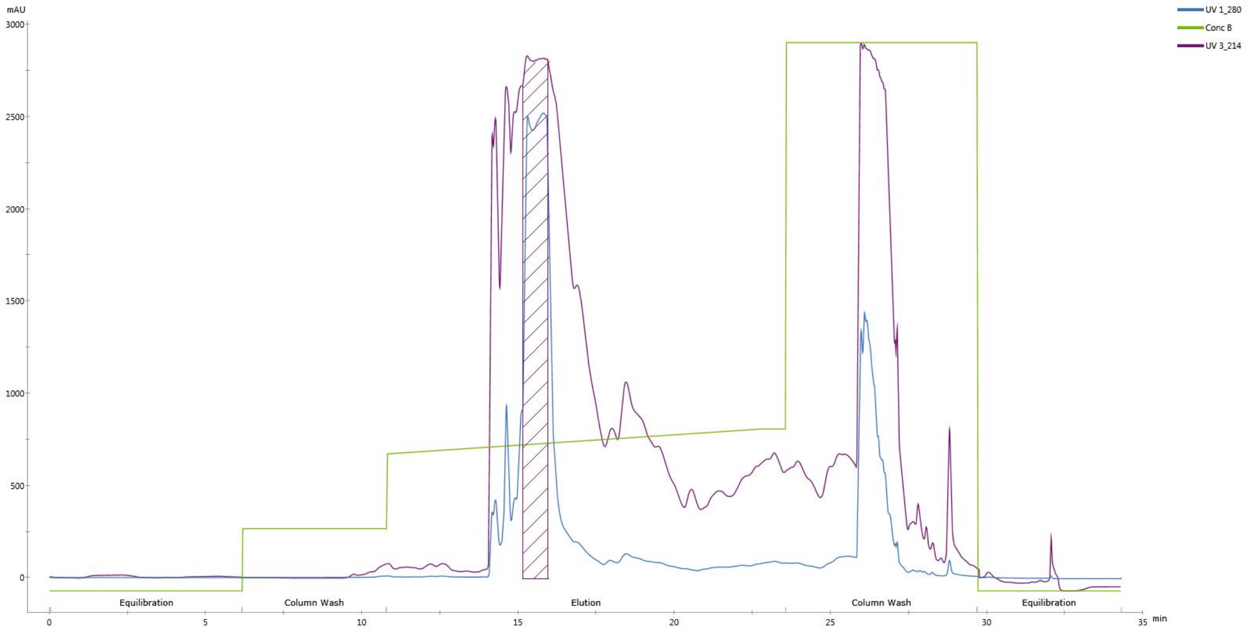

C

Peptide name	A2R3P7
Peptide sequence	EKGRRRWIFYFF
Molecular weight	1705
Resin type	Fmoc-Rink amide aminomethyl-polystyrene resin (ABCR GmbH & Co KG, Karlsruhe, Germany); loading capacity 0.78 mmol/g

Figure S19. Datasheet of peptide A2R3P7. A - Mass spectrum of peptide A2R3P7. B - Chromatogram of preparative HPLC purification of peptide A2R3P7. Chromatogram showing the gradient profile (green), UV absorbance at 280 nm (blue) and 214 nm (violet), with the target peak indicated by the shaded violet region. C- Characterization of peptide A2R3P7.

A

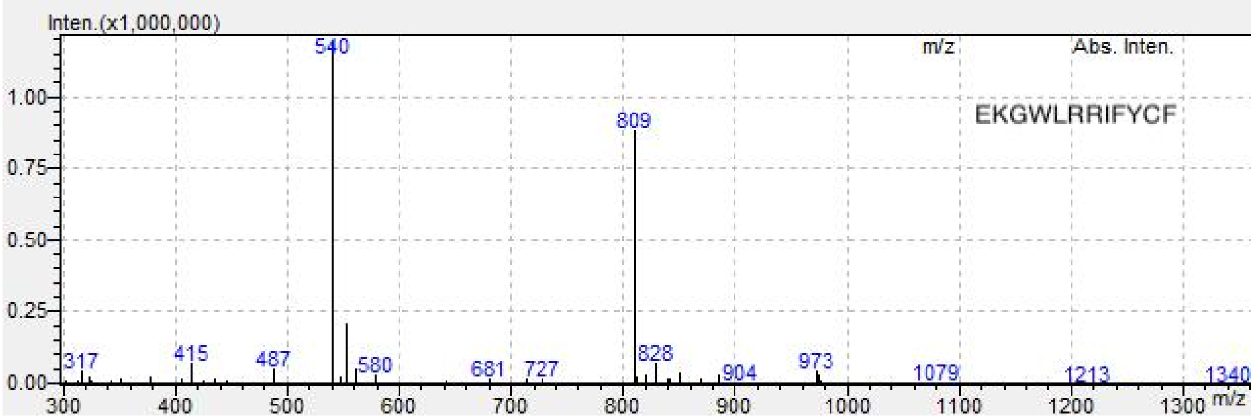

B

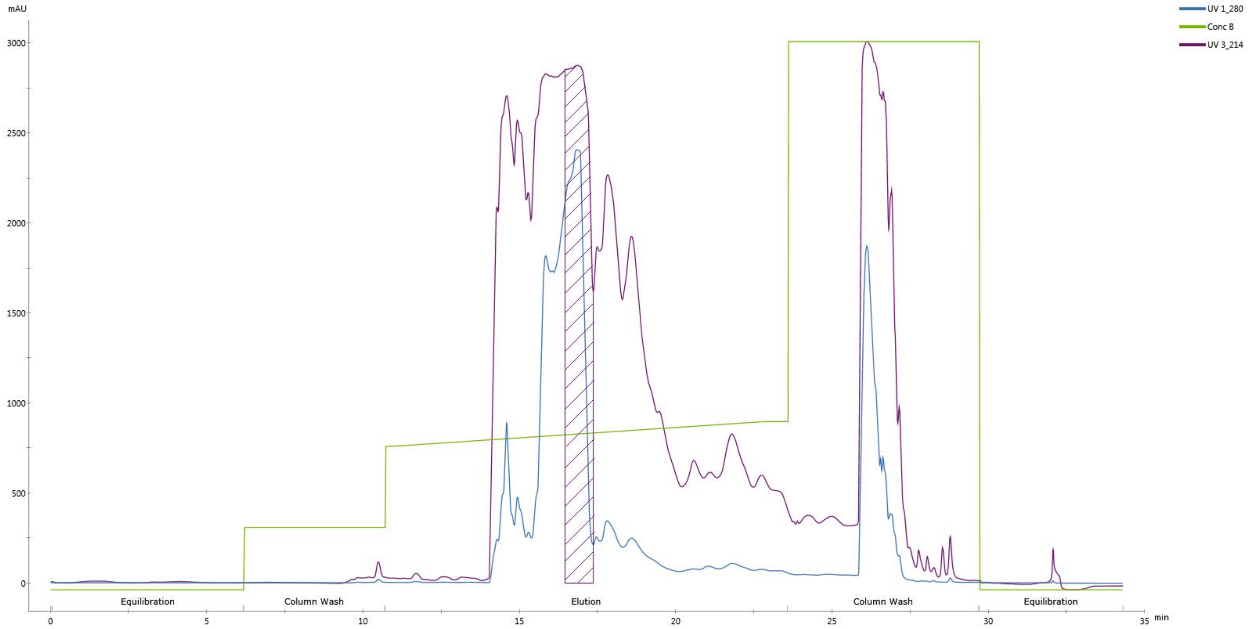

C

Peptide name	A2R3P8
Peptide sequence	EKGWLRRIFYCF
Molecular weight	1618
Resin type	Fmoc-Rink amide aminomethyl-polystyrene resin (ABCR GmbH & Co KG, Karlsruhe, Germany); loading capacity 0.78 mmol/g

Figure S20. Datasheet of peptide A2R3P8. A - Mass spectrum of peptide A2R3P8. B - Chromatogram of preparative HPLC purification of peptideA2R3P8. Chromatogram showing the gradient profile (green), UV absorbance at 280 nm (blue) and 214 nm (violet), with the target peak indicated by the shaded violet region. C- Characterization of peptide A2R3P8.

A

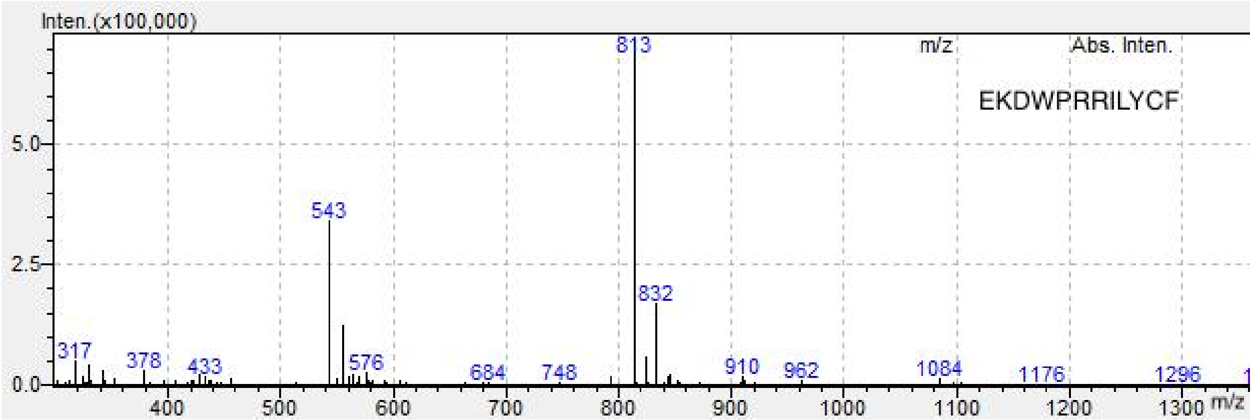

B

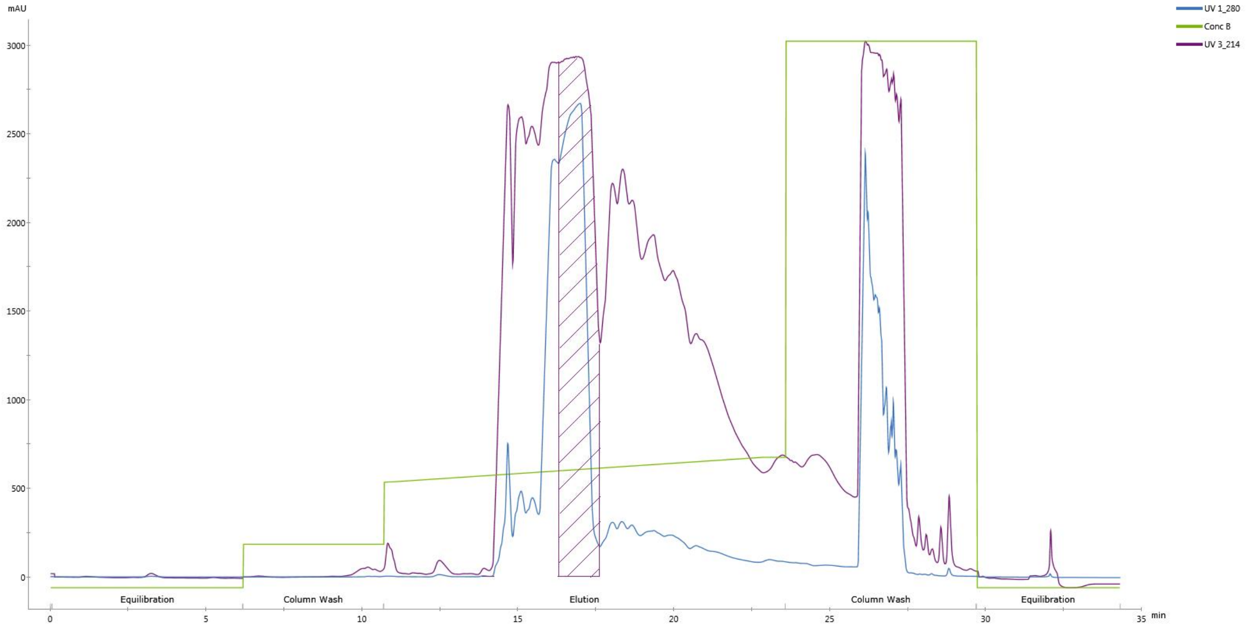

C

Peptide name	A2R3P10
Peptide sequence	EKDWPRRILYCF
Molecular weight	1626
Resin type	Fmoc-Rink amide aminomethyl-polystyrene resin (ABCR GmbH & Co KG, Karlsruhe, Germany); loading capacity 0.78 mmol/g

Figure S21. Datasheet of peptide A2R3P10. A - Mass spectrum of peptide A2R3P10. B - Chromatogram of preparative HPLC purification of peptide A2R3P10. Chromatogram showing the gradient profile (green), UV absorbance at 280 nm (blue) and 214 nm (violet), with the target peak indicated by the shaded violet region. C- Characterization of peptide A2R3P10.

A

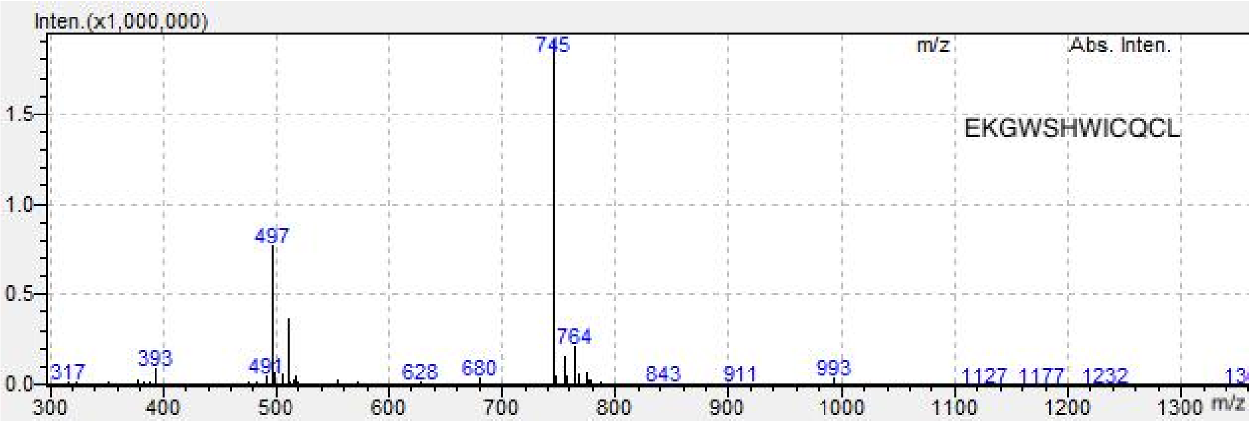

B

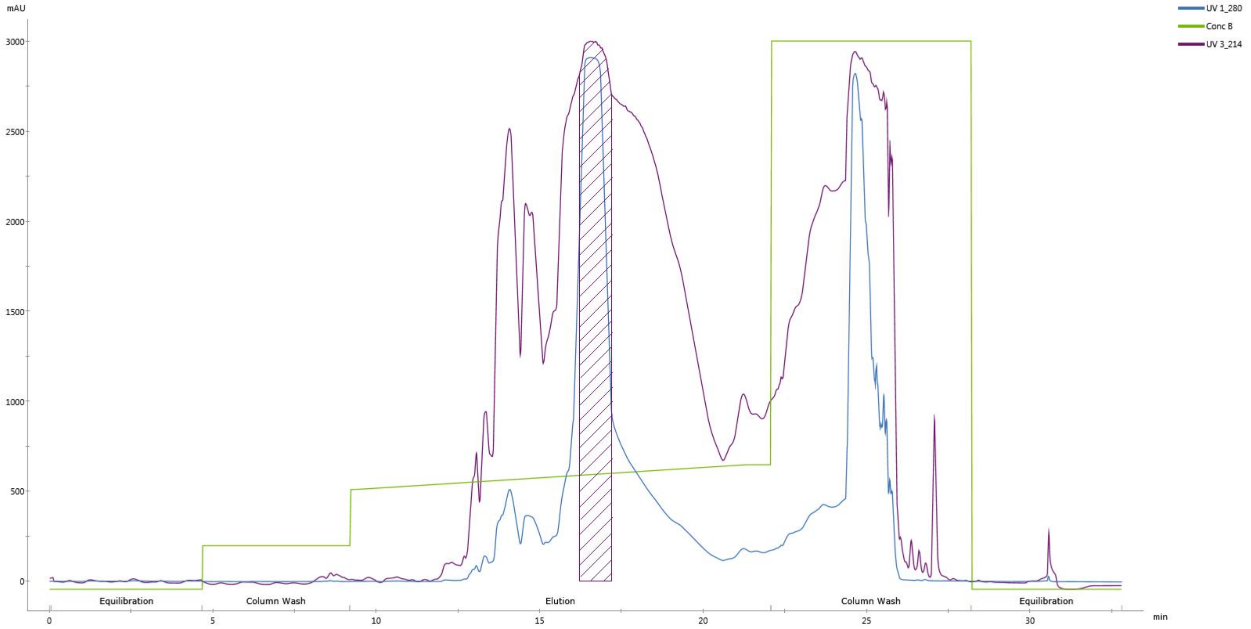

C

Peptide name	A2R3P11
Peptide sequence	EKGWSHWICQCL
Molecular weight	1490
Resin type	Fmoc-Rink amide aminomethyl-polystyrene resin (ABCR GmbH & Co KG, Karlsruhe, Germany); loading capacity 0.78 mmol/g

Figure S22. Datasheet of peptide A2R3P11. A - Mass spectrum of peptide A2R3P11. B - Chromatogram of preparative HPLC purification of peptide A2R3P11. Chromatogram showing the gradient profile (green), UV absorbance at 280 nm (blue) and 214 nm (violet), with the target peak indicated by the shaded violet region. C- Characterization of peptide A2R3P11.

A

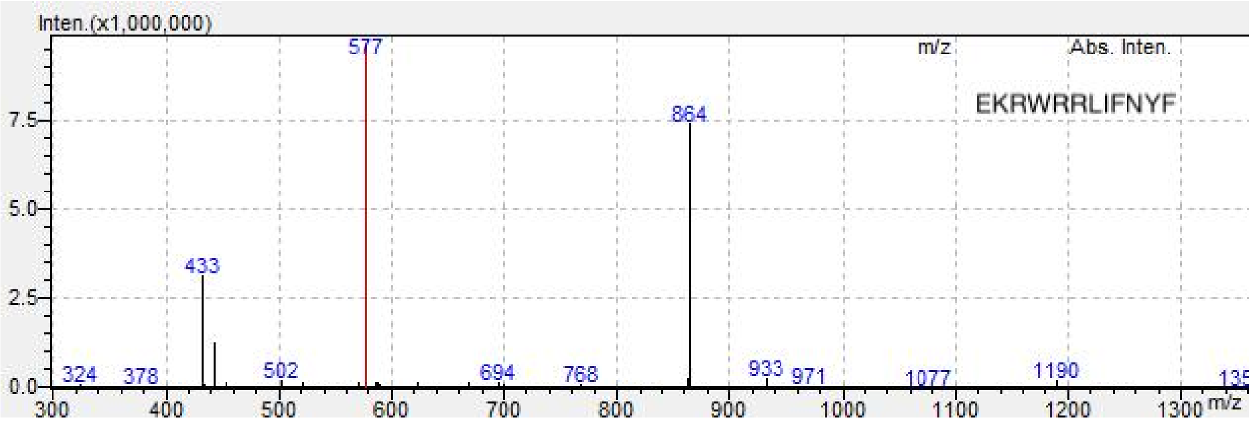

B

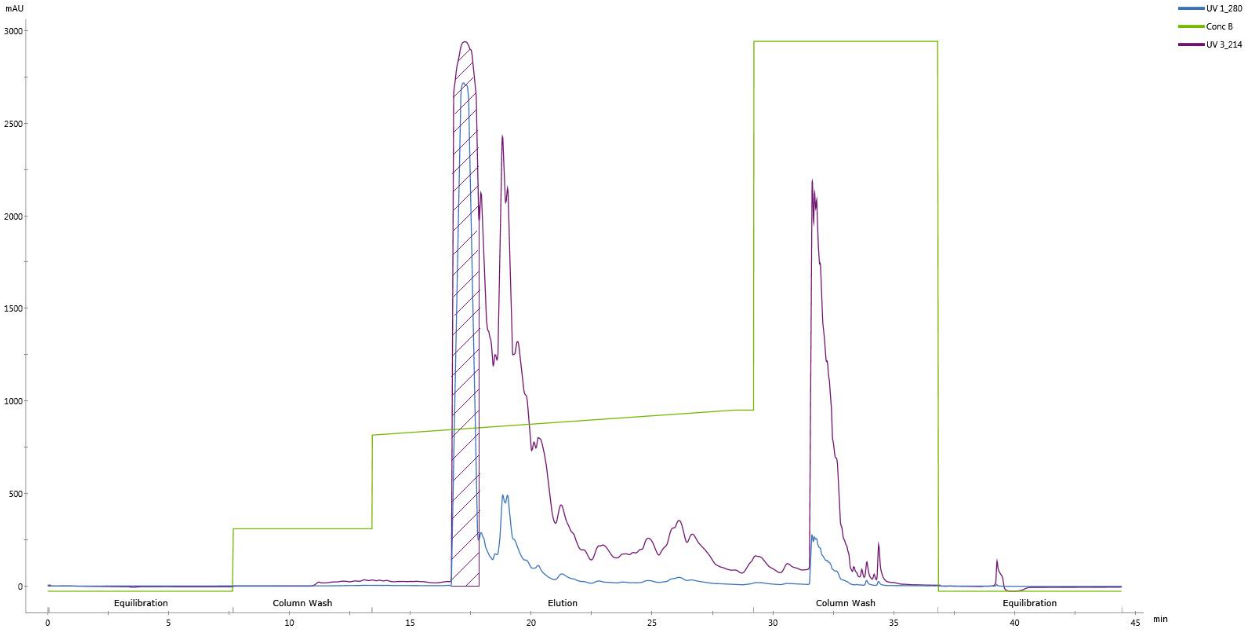

C

Peptide name	Hm-AMP2
Peptide sequence	EKRWRRLIFNYF
Molecular weight	1728
Resin type	Fmoc-Rink amide aminomethyl-polystyrene resin (ABCR GmbH & Co KG, Karlsruhe, Germany); loading capacity 0.78 mmol/g

Figure S23. Datasheet of peptide Hm-AMP2. A - Mass spectrum of peptide Hm-AMP2. B - Chromatogram of preparative HPLC purification of peptide Hm-AMP2. Chromatogram showing the gradient profile (green), UV absorbance at 280 nm (blue) and 214 nm (violet), with the target peak indicated by the shaded violet region. C- Characterization of peptide Hm-AMP2.
